# A World of (In)difference? Social Inequalities Among Infants’ Causes of Death in Mid-nineteenth-Century Amsterdam

**DOI:** 10.1093/shm/hkae066

**Published:** 2024-11-21

**Authors:** Sanne Muurling, Peter Ekamper

**Affiliations:** Department of History, Art History and Classics, Radboud University Nijmegen, PO Box 9103, 6500HD Nijmegen, The Netherlands; Netherlands Interdisciplinary Demographic Institute (NIDI-KNAW), PO Box 11650, 2502 AR The Hague, The Netherlands

**Keywords:** infant mortality, causes of death, social inequality, nineteenth century, Amsterdam

## Abstract

The relationship between mortality and socioeconomic status is among the most debated topics within historical demography. This article scrutinises social disparities in infant mortality and its underlying mechanisms in mid-nineteenth-century Amsterdam. We apply two methods of survival analysis (Cox proportional hazard models and Fine-Grey competing risk models) on newly digitised individual-level cause-of-death data for infants born in 1856 combined with civil certificates and population register data. Through a comparison of all-cause and cause-specific mortality, we bring to light important social differences in infants’ mortality risks; hazard ratios for congenital and birth disorders during early post-neonatal infancy were over 50 per cent lower for Amsterdam’s middle class than for unskilled workers. We argue that the social differentiation in infant mortality reflects stark intra-urban disparities in maternal health across social groups as well as a degree of medical ineffectiveness or even indifference structured along the same socioeconomic lines.

Dramatic changes in mortality and life expectancy are considered to be among the crowning achievements of the past 200 years.^1^ One of the principal drivers of this increased life expectancy and, with it, the first demographic transition, was the decline of infant mortality. Whereas less than 1 per cent of today’s deaths consist of those younger than 20 years old, around 1850, nearly a third of all deaths consisted of infants below the age of one.^2^ Together with a shift in the leading causes of death, the reduced share of infant deaths signals a substantial change in the very structure of mortality since the middle of the nineteenth century. It is difficult to overstate the enormity of this development.

Still, this health transition was not necessarily a process that was equally distributed across societies. Differences between regions, socioeconomic status and cultural factors such as religion and gender are believed to have crafted different pathways to low mortality regimes, though their exact configurations have remained open to debate. Patterns of infant deaths pose a fascinating conundrum within ongoing scholarly debates about the roots and long-term development of health inequalities. While infant mortality nowadays commonly serves as an indicator of socioeconomic well-being, scholars are undecided about the association between social status and infants’ risk of dying in periods prior to the epidemiological transition. Important syntheses of the past decade and a half lean towards the premise that the majority of infant deaths were unavoidable and that mortality was consequently largely invariant between social groups prior to the epidemiological transition.^3^ This proposition is supported by evidence from a range of studies on Iceland, Germany, France, England and Sweden that – mainly based on all-cause infant mortality data – found no indications that social status conferred survival advantages for infants, at least not until the end of the nineteenth century.^4^ However, this view is contradicted by findings from Switzerland, Italy, as well as England and the USA, which did demonstrate significant socioeconomic divergence during earlier periods as far back as the seventeenth century.^5^ While these disparate findings call into question the universality of an association between socioeconomic status, they also emphasise the urgency of a closer examination of the underlying mechanisms behind social differentials across time and space.

To further our insights into the roots and historical evolution of mortality inequality, Janssens and others have recently emphasised the importance of a cause-specific approach.^6^ In general, socioeconomic variation in infant mortality can arise from the uneven distribution of risk and protective factors between groups.^7^ The mechanisms through which social class could affect infant health operated at the individual, household and even broader community level, encompassing aspects such as maternal age at birth, nutritional deficiency, personal illness control and injury, as well as household crowding and access to clean water.^8^ Because categories of deciding factors are known to differ across the first year of life, scholars may draw some inferences about the role of certain determinants through the age at death, even when they only have all-cause mortality data at their disposal.^9^ However, cause-of-death data provides additional, more fine-grained means to interrogate mortality disparities since causes of death themselves are contingent on different risk factor mechanisms.^10^ Disease categories therefore provide additional clues as to what the causes of health advantage may be, which allows us to empirically verify assumptions about the prevalence of certain mortality determinants throughout the first year of life. Moreover, the use of a disease-specific approach enables us to uncover the presence of social inequalities for certain diseases, even when they are not driving an overall statistically significant social gradient at the all-cause level. As such, cause-specific analyses may provide deeper insights into the reasons for emerging differentials in infant mortality throughout different stages of the epidemiological transition.

This study takes on this challenge by making use of the newly digitised individual-level cause-of-death data for Amsterdam. It does so through an intra-urban analysis of mortality of infants born in 1856: currently the first year for which we possess individual-level records from the cause-of-death registration implemented around this time. While the Netherlands is known as the ‘first modern economy’, the early to mid-nineteenth century must be viewed as a period of stagnation.^11^ Mid-nineteenth-century Amsterdam represents an urban setting prior to later large-scale demographic and epidemiologic transitions, as well as the sanitary revolution, when infant mortality was still sky-high and infectious diseases were omnipresent. How did different individual and environmental-level exposures affect infant mortality risks due to different causes across the city two decades *before* the onset of these transformations? To what extent did they result in a social differentiation in infants’ risk of dying?

To scrutinise the existence and mechanisms of social inequalities among infant mortality in mid-nineteenth-century Amsterdam, we will first discuss how a cause-specific and intra-urban analysis provides an important contribution to the historical debates concerning socioeconomic differentials. We then consider how Amsterdam in 1856 can be considered a city at a standstill in terms of demography, mortality, social geography, sanitation and medical care. This is followed by sections about the sources and methods used in this study. Utilising two methods of survival analysis (Cox proportional hazard models and Fine-Grey competing risk models), we scrutinise the newly available Amsterdam cause-of-death data, first for all-cause and then for cause-specific mortality.^12^ We argue that our approach brings to light a social differentiation in mortality that reflects both intra-urban health disparities and medical indifference in recording deaths structured along the same socioeconomic lines.

## Socioeconomic Differentials in Historical Infant Mortality

The relationship between mortality and socioeconomic status is among the most debated topics in the field of historical demography over the past few decades. A central question in recent literature is whether social differences in death persisted over a broad historical period and a variety of places (the constancy hypothesis) or whether they varied over time, alternating between periods of increasing gaps in life expectancy between different social groups and periods of convergence.^13^ According to the divergence-convergence hypothesis, the predominance of infectious diseases and limited medical knowledge produced low levels of inequality before the seventeenth century.^14^ The following two centuries, inequalities would start to diverge due to an improvement in the standard of living of the well-to-do classes and a stagnation or worsening thereof among the lower classes, followed by a convergence from the mid-nineteenth century onwards caused by the diffusion of medical, sanitation and economic advances among the population.

Proponents of the constancy or fundamental cause theory (FCT) conversely suggest that inequalities in accessing resources provided a persistent advantage to higher socioeconomic groups over historical time.^15^ A recent extension of this theory by Clouston et al. recognises that socioeconomic inequalities fluctuated historically by disease category.^16^ They suggest that inequalities in health may not be observed simultaneously for all diseases and that cause-specific inequalities instead emerged and vanished over time. Due to the rise of new socially patterned diseases over time, a persistent socioeconomic gradient in all-cause mortality could be maintained even when mortality risks became more equal in formerly dominant groups of diseases.

Infant mortality has been a prominent object of scholarly scrutiny due to the pivotal role of this age group in bringing about the increase in life expectancy during the late nineteenth century. Findings from these studies examining the nature of the relationship between socioeconomic status and infant mortality in pre- or early transitional periods have been anything but conclusive. In recent years, Bengtsson, Dribe and others have been notable critics of the idea of early social divergence in mortality.^17^ Based on all-cause mortality for southern Sweden, they have shown that socioeconomic differentiation in post-neonatal (29 days to the age of 1) and child mortality (1–15 years) had only come into existence during the latter part of the nineteenth century, followed by the emergence of a social gradient in mortality among adults during the twentieth century.^18^ Even though the socioeconomic homogeneity of the area may call the generalisability of these findings into question, a range of studies on other places elsewhere in Europe similarly found a clear association to be lacking in pre-transitional periods.^19^

At the same time, other scholars did observe significant socioeconomic differentiation in infant mortality during earlier periods as far back as the seventeenth century, though the early and mid-nineteenth century are better documented.^20^ For example, Mühlichen observed that the risk of death among infants in medium- and low-status groups in Rostock in the period 1815–29 was between 65 and 89 per cent higher than the risk of death among their high-class counterparts.^21^ In Venice, during the period 1850–59, the risk of death during the first 6 months of life for the children of day labourers exceeded the risks of death among the children of wage workers, artisans and shopkeepers, and the middle classes by 20, 28 and 82 per cent, respectively.^22^

Three important conclusions may be drawn from these disparate scholarly findings. First, there does not seem to have been a universal association between socioeconomic status and infant mortality across time and space.^23^ Second, the emergence of significant social differentiation cannot simply be reduced to a by-product of modernisation.^24^ Third, the empirical assessment of the underlying mechanisms is pertinent to understanding the highly context-specific roots and evolution of infant mortality patterns.

Disagreements on disparities in infant mortality dovetail with uncertainties about underlying causal mechanisms. One of the most commonly used theoretical frameworks for determinants affecting infant mortality is provided by Mosley and Chen.^25^ According to this framework, all social and economic determinants had to operate through (one or more of) five variables to affect child survival: (i) maternal factors such as age and birth interval, (ii) environmental contamination, (iii) nutrient deficiency of child or mother, (iv) injury and (v) personal illness control.^26^ The latter category plays a crucial role in the recent work by Clouston et al., who view the differential diffusion of new knowledge and health technologies to prevent or cure disease as the core driver of inequality during the epidemiological transition.^27^ Despite their emphasis on the role of intentional human modification, they also concede that ‘natural’ or unintentional variation in cultural or health-related lifestyles and differences in access to resources such as living circumstances and nutritional status could produce significant mortality inequalities.^28^

An excellent recent overview of the discussion of these individual factors and their applicability to the historical setting has been provided by Murkens et al.^29^ It therefore suffices to point out several factors commonly mentioned as potentially producing inequalities that were *absent* in most Western societies prior to the epidemiological transition. Notably absent still were the sanitary advances such as piped water and sewerage, which are generally believed to have contributed to the lowering of infant mortality levels in the long run.^30^ Access to knowledge and to medical technologies is another relevant factor. Before medical knowledge became widely diffused, only certain groups with superior resources could have access to or act upon it, giving rise to mortality inequalities.^31^ However, the influence of this factor is presumed to have been limited in a historical context where medical knowledge could not yet save infants once they had fallen severely ill.^32^ This means that the list of possible determinants responsible for infant mortality disparities before the epidemiological transition is most likely reduced to factors like maternal health, breastfeeding practices and housing conditions – though further empirical analysis is needed to test these assumptions.

An important way to query the determinants of infant mortality patterns is through the examination of cause-of-death categories. Traditionally, scholars have made use of the age at death to draw inferences about causal mechanisms from all-cause infant mortality data. Survival time of infants is telling because categories of deciding factors shifted across the first year of life, as mechanisms related to the development of the foetus during the mother’s pregnancy and problems during childbirth dominating mortality during the first 28 days gave way to environmental factors after this time.^33^ During recent decades, the field of historical demography has witnessed a surge of interest in individual-level causes of death as a way to enhance our understanding of changes in mortality patterns and their underlying determinants.^34^ Because causes of death are contingent on different risk factor mechanisms, disease categories provide additional and possibly more fine-grained indications of the underlying causes of health mechanisms than an examination of age at death alone.^35^ The examination of mortality risks for isolated diseases or disease groups furthermore enables us to capture inequalities even if they are concealed at the all-cause mortality level. As such, a cause-specific approach expresses the potential impact of socioeconomic status more clearly than an analysis of all-cause mortality could do. Individual-level cause-of-death data are also key to the European SHiP+ network which brings together historical demographers, medical historians and historical epidemiologists to study health changes over time and space.^36^ The construction of joint coding and classification schemes facilitates comparative work within and between European countries over long periods of time. As a society on the cusp of fundamental change, this cause-specific inquiry into infant mortality in mid-nineteenth-century Amsterdam therefore provides the groundwork for future studies of the long-term changes in the distribution of health across society.

## Mid-nineteenth-Century Amsterdam

In the mid-nineteenth century, Amsterdam’s inhabitants could not yet foresee that they stood on the eve of large-scale changes that would transform the demographic, socioeconomic and health contours of their lives. Around this time, the city ranked among the top 20 largest cities in Europe, but with its approximately 225,000 inhabitants, Amsterdam had just barely regained its pre-economic decline population size from a century earlier.^37^ After 1860, an economic revival and accompanying increase in employment opportunities attracted a steadily growing number of migrants to the city, with a peak during the 1870s and 1880s. Combined with the birth surplus, Amsterdam’s population swelled considerably during this period.^38^ By the end of the nineteenth century, the city had seen its population double to more than 510,000 inhabitants. Compared to what the city had been and what it would become, it is not surprising that mid-nineteenth-century Amsterdam is commonly characterised as a city at a standstill.^39^

This stagnation extended to a wide range of other aspects of life. Like many other western European capitals, Amsterdam still possessed its early modern shape consisting of concentric shells encased by ramparts (see Supplementary Figure S1 for a visualisation of the city’s neighbourhoods). Some 2,000 inhabitants resided in the extramural outskirts, but the overwhelming majority of the population lived within the densely populated confines of the city walls that were only torn down during the last quarter of the nineteenth century to accommodate the first urban expansion since the seventeenth century. Especially crowded was the poorer Jordaan neighbourhood on the western border of the city, the harbour area and the so-called Jewish neighbourhood southeast of the city centre.^40^ In the following decades, a host of medical doctors and social reformers would express severe concern about the slum dwellings in these areas.^41^ Rooted in ideals of civilising the lower classes, philanthropic housing organisations started building and improving houses in the poorest parts of Amsterdam during the second half of the nineteenth century.^42^ Structural improvements for larger parts of society only came after 1901, though, when a new national bill made the construction and inhabitation of good and healthy housing a government concern.

Social inequalities also manifested themselves in the arenas of health and mortality. Infant mortality is widely used as an indicator of trends in mortality and population health in general.^43^ Infant mortality rates (IMRs) were high during most of the nineteenth century. According to the calculations by Ekamper and van Poppel, around 200 per 1,000 girls born alive in 1851 succumbed within their first year, as did 235 boys.^44^ In a spatial sense, these IMRs by and large followed the population density patterns: overall, they were relatively low in the thinly populated richer parts of the city and particularly high in the densely populated poorer areas of the city. The Jewish neighbourhoods constituted an interesting exception to the general rule, with low IMRs in spite of their infamous poverty and crowdedness.^45^ On a city level, any real decline only set in after the 1880s, dipping below a rate of 100 around 1910.

What caused the late-nineteenth-century infant mortality decline remains subject to scrutiny. Internationally, various scholars have emphasised the importance of sanitary improvements such as clean water supply, waste disposal and sewerage.^46^ The issue of drinking water supply had already been raised in the sixteenth century in connection with the increased pollution of Amsterdam’s canals, which were commonly used for household purposes by the poor as well as for the disposal of waste.^47^ Drinking water was also transported to Amsterdam from neighbouring regions, but the poor remained largely dependent on the water from the canals and rain barrels.^48^ In 1851, the city authorised the construction and commercial exploitation of a water supply system. From 1854 onwards, the *Duinwatermaatschappij* (Dune Water Company) started distributing clean tap water to a rapidly increasing number of ‘subscribed’ private houses connected to the expanding network of waterpipes across the city, though even over a decade later large sections of Amsterdam’s population were still unable to afford a connection to the tap water system.^49^ Before 1880, there were no systematically organised cleaning and waste disposal services to speak of, and a city-wide sewer system was not built until 1906.^50^ Amsterdam in the mid-nineteenth century therefore stood at the cusp of pivotal sanitary improvements, but the overwhelming majority of its inhabitants still lived their lives without them.

Finally, in terms of medical care and knowledge, the mid-nineteenth century also still marks a period before marked change. Even though medical literature of the time had started to zero in on the importance of breastfeeding for infant survival chances, it is unclear to what extent this knowledge was disseminated beyond the learnt journals.^51^ Woods, Løkke and van Poppel noted the impact of the introduction of trained midwives and the quality of obstetric care on infant mortality rates in nineteenth-century Europe.^52^ If any obstetric practitioner assisted in the delivery at all in the nineteenth-century Netherlands, it was usually the midwife.^53^ At the time, midwives in the Netherlands were relatively well trained as compared to many other countries, such as Britain and the USA.^54^ Preventative care for infants and young children was largely unknown during most of the nineteenth century. Only after 1900 did new paediatric insights among physicians spur the establishment and rapid expansion of medical services and facilities in this field in Amsterdam.^55^ From the perspective of demography, urban planning, sanitary infrastructures and mortality patterns, children born in 1856 cannot yet be seen as beneficiaries of the significant improvements that would befall their successors. Half a century later, the city would have been rendered unrecognisable to the children born in the mid-nineteenth century, had they lived long enough to experience it.

## Sources

The main sources used in this study of infant mortality are the Amsterdam cause-of-death database (hereafter ACD) and birth and death certificates.^56^ The ACD is based on individual cause-of-death registers initiated by the city authorities in 1853. New municipal rules stated that all deaths needed to be verified by a doctor before burial could take place and, swayed by the members of Amsterdam’s Medical Practitioners’ Society (the *Geneeskundige Kring*), demanded a verification of the cause of death before a death certificate could be issued.^57^ This resulted in a systematic registration of all deceased individuals and their causes of death in Amsterdam from 1854 until 1940. While diagnostic practices likely improved in quality over time, especially following new nationwide legislation on cause-of-death registration in 1865, the earlier registers are considered to have been fairly reliable in terms of registration consistency and diagnostic accuracy.^58^ After all, Amsterdam’s *Geneeskundige Kring* was very prominent in the Dutch Hygienist movement and stood at the forefront of medical knowledge in the Netherlands at that time. The cause-of-death registers for the years 1854 and 1855 were incomplete or lost, making 1856 the first year suitable for analysis. In addition to the cause of death, the registers record the residential address of the deceased, the date of death, age, sex, marital status and occupation.

From the birth registers, we selected the birth certificates of the cohort of all children born in the year 1856 and tracked their survival up to their first birthday by linking them to the death certificates and the cause-of-death records of the years 1856–57. The civil certificates provided information about the child’s name, their birth date, whether there were multiple births, as well as the parents’ names, their marital status, occupations, age of the father and residential address. Linking the cause-of-death data to the civil certificates also enabled us to correct for missing or unidentified births and duplicate registrations and to calculate exact ages at death from the dates of birth and death. Additional information was gathered from the population registers of the slightly earlier period 1851–53.^59^ Enforced in the Netherlands by the Royal Decree of 1849, the population registers combine census listings with vital registration for the entire population of a municipality with the household as the registration unit.^60^ Through the population register, we were able to add to the dataset the dates of birth of the father and mother as well as religious affiliation for over 80 per cent of infants from the 1856 birth cohort.

Cadastral maps and corresponding cadastral registers available from the historical geographical information system Amsterdam (HISGIS Amsterdam) were used to link the residential addresses to the tax value of the dwellings as an indication of the quality of the housing.^61^ The historical addresses were assigned an exact geographical location (longitude and latitude) using the HISGIS Amsterdam location points of 1853.^62^ From these sources, we have also computed proximity to surface water (canals) and determined whether a house was located in a backstreet alley. Information on midwives has been extracted from the historical address books, complemented by information from 1851 to 1853 population register.^63^ The lack of institutional public health facilities meant that the majority of Dutch women gave birth to their children at home, regardless of their socioeconomic status. If anyone assisted in the delivery at all, it was usually midwives.^64^ We have therefore linked the historical addresses to data on midwives, allowing us to determine geographical proximity of infants to these health practitioners.

Our total dataset includes 8,751 infants born in 1856, 2,201 of whom died within their first year of life (including 502 stillborn). For our analyses of infant mortality, we have left out a small number of children not born at a permanent address (16 foundlings, 7 of whom were stillborn and 23 children born on board of ships temporarily docked in Amsterdam, of whom 4 died within their first year of life), as well as all other 495 stillborn children. As such, the remaining dataset includes 8,217 live births in 1856, 1,695 of whom died within their first year of life. A total of 1,598 of these deaths (94 per cent) could be linked to the cause-of-death records by matching the date of death, sex and residential address.^65^ The remaining 97 deaths were classified as unknown causes of death. Additionally, we were able to link 6,611 birth records (81 per cent) to one or both parents in the Amsterdam 1851–53 population register with additional data on religious affiliation and dates of birth of the parents by matching combinations of names, ages, occupations and residential addresses of the parents.^66^ As for the residential addresses, we have an overall linkage rate of over 99 per cent for all live births. For 19 infants (0.2 per cent), the address was lacking from all registers, and for 1 infant we were unable to distinguish where the address in the outer areas exactly was. For 14 infants (0.2 per cent) we estimated the location using partial addresses (based on midpoints of the residential street).

The Dutch civil registry is considered to have been of high quality and by and large complete.^67^ The precise administrative system meant that the recorded vital events are likely to have represented population vital events well, with the notable exception of family ties that did not leave a paper trail and those that were not in accordance with the law. This particularly affected pre- and extramarital children. In our dataset, we lack information on the names of fathers of 621 infants (7.5 per cent), which aligns with the percentage of infants born out of wedlock for that year.^68^

## Methods

To analyse the influence of newborns’ characteristics on their mortality risk, we make use of two methods of survival analysis: Cox proportional hazard models and Fine-Grey competing risk models. First, to scrutinise all-cause infant mortality, we applied Cox proportional hazard models to the live births from 1856.^69^ This is a model commonly used to estimate the effects of covariates on the hazard of the occurrence of the event, in this case infant death. We calculated survival time measured in days from the date of birth up to the date of death or, for those surviving their first year of life, up to their first birthday. Research into infant mortality has indicated that deciding factors differed across the first year of life.^70^ While mortality during the first 28 days after birth is largely determined by the development of the foetus during the mother’s pregnancy and problems during childbirth, environmental factors are believed to gain in importance after the first month of life.^71^ The size of our sample is too small for subdivision into the age category of perinatal mortality. We therefore opted for a multi-episode model for mortality in the neonatal period (first 28 days of life), ‘early’ post-neonatal infancy (from day 29 up to the 6^th^ month of life), and ‘later’ post-neonatal infancy (from the 7^th^ up to the 12^th^ month of life).

We then applied Fine-Grey competing risk models to explore survival chances for specific causes of death.^72^ As the name indicates, this model was explicitly developed to take competing risks into account, providing a better estimation of hazard ratios (HR) due to one cause in the presence of other possible causes of death. For instance, if one seeks to study infant mortality due to infectious diseases, death attributable to a non-infectious cause serves as a competing event. After all, an infant who died from measles was no longer at risk of death from jaundice.

The following variables were included in our models: age of the child in days, sex of the child, age of the mother at birth, age difference with age of the father, whether the mother was single, whether the infant was part of a multiple birth, religion, social class, season of birth, whether the child was living in a backstreet alley, the tax value of the dwelling, the water quality of the canals, whether the house was located in front of a canal, distance to the nearest midwife and neighbourhood district. The variables religion, social class and neighbourhood district require some clarification. Religious affiliation was extracted from the father’s entry in the population register, or if the father was unknown, from the mother’s entry. These registers originally recognised 15 categories, which we aggregated into 4 broader religious groups (Dutch Reformed, Other Protestant, Catholic and Jewish).^73^ Social class consists of five categories (*unskilled workers* like day labourers or seamen, *semi-skilled workers* such as basket makers or weavers, *skilled workers* like blacksmiths or bakers, *middle class* like clerical workers or shopkeepers and *elite* like medical doctors or nobility) based on the Social Power scheme applied to the father’s occupation or that of the mother if the father is unknown.^74^ We have chosen to employ this social classification scheme rather than creating our own to enable comparisons across national boundaries and time. Neighbourhood district provides an additional variable to explore any notable spatial effects not sufficiently captured by individual characteristics of the deceased. Based on Laloli’s subdivision of mid-nineteenth-century Amsterdam, the city’s 50 administrative neighbourhoods are classified into 8 largely socio-economically homogenous districts (see Supplementary Figure S1).^75^Table 1 presents the descriptive statistics of our dataset of all children born in Amsterdam in 1856.

**Table 1.** Descriptive statistics of children born in Amsterdam in 1856

Variable	Total	Per cent	IMR	Variable		Total	Per cent	IMR
Sex				Tax value residential address				
Females	4,037	49.1	194		<100	2,446	29.8	233
Males	4,180	50.9	218		100–200	3,281	39.9	207
Age mother					200+	2,470	30.1	175
15–19 years	103	1.3	184		Unknown	20	0.2	600
20–24 years	497	6.0	137	Backstreet alley				
25–29 years	1,512	18.4	177		Yes	1,152	14.0	240
30–34 years	2,095	25.5	183		No	7,046	85.7	200
35–39 years	1,480	18.0	197		Unknown	19	0.2	632
40+ years	665	8.1	226	Distance to nearest midwife				
Unknown	1,865	22.7	276		<75 m	2,112	25.7	210
Age difference father-mother					75–150 m	2,622	31.9	188
Younger (2+ years)	1,028	15.4	187		150–300 m	2,865	34.9	209
Equal (−2 to +2 years)	1,620	24.2	184		300–600 m	536	6.5	241
Older (2–9 years)	1,776	26.4	182		600+ m	62	0.8	290
Much older (10+ years)	619	9.2	186		Unknown	20	0.2	600
Unknown	1,479	24.8	274	Water quality canals				
Multiple birth					Seafront	1,910	23.2	194
Yes	240	2.9	533		‘Best’	261	3.2	199
No	7,977	97.1	196		‘Intermediate’	2,776	33.8	189
Religion					‘Worst’	3,250	39.6	226
Dutch reformed	3,320	40.4	192		Unknown	20	0.2	600
Other protestant	1,147	14.0	179	House in front of canal				
Catholic	1,433	17.4	210		Yes	2,245	27.3	188
Jewish	709	8.6	117		No	5,952	72.4	212
Unknown	1,608	19.6	292		Unknown	20	0.2	600
Social class				Neighbourhood				
Elite	151	1.8	146	1	Centre	1,874	22.8	210
Middle class	1,852	22.5	172	2	Jewish quarter	804	9.8	150
Skilled workers	2,618	31.9	185	3	Islands	904	11.0	217
Semi-skilled workers	947	11.5	189	4	Jordaan	2,076	25.3	231
Unskilled workers	1,929	23.5	214	5	Northern canals	616	7.5	156
Unknown	720	8.8	388	6	Southern canals	1,020	12.4	202
Single mother				7	Plantage Weesper	694	8.4	183
Yes	609	7.4	489	8	Outer areas	210	2.6	310
No	7,608	92.6	184	9	Unknown	19	0.2	632
Season of birth								
Spring	2,211	26.9	198					
Summer	1,872	22.8	202					
Autumn	1,998	24.3	214					
Winter	2,136	26.0	212	Total		8,217	100.0	206

*Note*: IMR = infant mortality rate.

*Source(s)*: Own calculations based on linked data from Amsterdam cause-of-death database (ACD), Amsterdam birth (1856) and death (1856–57) certificates, Amsterdam population register 1851–53 and HISGIS Amsterdam.

Lastly, for the competing risk analysis, the causes of death are divided into 10 causal categories according to the joint coding scheme ICD10h constructed by the SHiP+ network to study mortality dynamics using individual cause-of-death data across Europe between 1850 and 1950 in a systematic way.^76^ The coding scheme is sensitive to historical terminology; by coding words rather than diseases, it seeks to avoid undesirable levels of interpretation of disease descriptions. The ‘infantcat’ classification within this scheme has specifically been created by experts in the SHiP+ network to study infant mortality in a historically sensitive way, having checked for appropriate seasonality patterns when relevant and keeping separate ‘controversial’ causes such as convulsions to allow for further testing.^77^ Based on aetiology, it distinguishes infectious diseases based on their mode of transmission: water- and foodborne infectious diseases (such as abdominal tuberculosis, cholera, diarrhoea, gastroenteritis, peritonitis and typhoid fever), airborne infectious diseases (bronchitis, diphtheria, influenza, measles, meningitis, pneumonia, scarlet fever, tuberculosis and whooping cough) and other infectious diseases (nephritis, sepsis, syphilis and tetanus). It furthermore distinguishes deaths caused by endogenous factors such as congenital and birth disorders (category 4), debility (category 5), convulsions (category 6), teething (category 7), other natural causes (category 8), non-natural causes (category 9) and unknown causes of death (category 10).

## All-Cause Mortality

In 1856, the overall infant mortality rate in Amsterdam was 206 per 1,000 live births. This was about 7 per cent higher than the national average of that year.^78^ Amsterdam’s above-average mortality rates match both the higher levels of infant mortality in the 1840s and 1850s found in the entire Western part of the country and the higher urban levels compared to rural levels.^79^ Poor housing and working conditions, sanitary conditions, ease of transmission and higher exposure to infectious disease typical of urban areas have commonly served as explanations for a widespread ‘urban penalty’ in historical Europe.^80^

To explore differentials in the risk of infant mortality *within* the city of Amsterdam, we employ Cox proportional regression models. The outcomes of these models are HR. When the HR is larger than one, the predictor is associated with an increased risk or, in other words, with decreased survival. Conversely, a hazard ratio lower than one is associated with improved survival chances. We first examine various sociodemographic variables such as the infant’s sex, age of the mother, whether the infant is part of a multiple birth, the mother’s marital status, occupational status and religious affiliation in univariate models for each variable separately (Table 2, model 1) and compare them to a multivariate model including all variables simultaneously (model 2). This analysis reveals a similar differentiation of sociodemographic characteristics in infant mortality as found for Amsterdam in 1851: there was a significant overall mortality disadvantage for male infants, for multiple births and for births to an older mother.^81^ Similar disadvantages have been observed by other studies, and it is likely that most of them were related to differences in gestational development and dissimilarities at the chromosomal or hormonal level.^82^

**Table 2.** Cox proportional hazards model HR for infant mortality by explanatory and control variables, Amsterdam, children born in 1856

		Univariate models		Multivariate model		Multivariate multi-episode models					
		(1)		(2)		(3)		(4)		(5)	
		Total		Total		Neonatal		Early post-neonatal		Later post-neonatal	
		HR	*p* > |*z*|	HR	*P* > |z|	HR	*p* > |*z*|	HR	*p* > |*z*|	HR	*p* > |*z*|
Sex	Males	1.152	0.004^***^	1.141	0.007^***^	1.291	0.027^**^	1.158	0.027^**^	1.019	0.839
Age mother											
15–19		1.364	0.232	1.222	0.446	0.232	0.159	2.034	0.024^**^	0.733	0.621
20–24		(ref)		(ref)		(ref)		(ref)		(ref)	
25–29		1.320	0.041^**^	1.203	0.178	0.818	0.495	1.364	0.104	1.213	0.472
30–34		1.361	0.019^**^	1.259	0.085^*^	0.857	0.587	1.202	0.329	1.782	0.023^**^
35–39		1.485	0.003^***^	1.385	0.018^**^	1.085	0.780	1.310	0.167	1.807	0.025^**^
40+		1.727	0.000^***^	1.692	0.000^***^	1.134	0.703	1.789	0.005^***^	1.966	0.020^**^
Unknown		2.204	0.000^***^	2.268	0.001^***^	1.195	0.719	2.847	0.005^***^	2.808	0.043^**^
Age difference father											
Younger (2+ years)		1.035	0.675	0.948	0.534	1.059	0.787	0.993	0.951	0.824	0.224
Equal (−2 to +2 years)		1.020	0.783	1.000	0.997	1.313	0.124	0.991	0.926	0.888	0.383
Older (2–9 years)		(ref)		(ref)		(ref)		(ref)		(ref)	
Much older (10+ years)		1.027	0.788	1.071	0.496	1.569	0.050^*^	0.875	0.353	1.214	0.272
Unknown		1.620	0.000^***^	0.599	0.005^***^	1.462	0.272	0.426	0.002^***^	0.526	0.089^*^
Multiple births		3.837	0.000^***^	3.929	0.000^***^	7.705	0.000^***^	3.508	0.000^***^	1.823	0.023^**^
Religion											
Dutch Reformed		(ref)		(ref)		(ref)		(ref)		(ref)	
Other Protestant		0.922	0.314	0.918	0.290	1.166	0.416	0.776	0.028^**^	1.082	0.582
Catholic		1.110	0.135	1.088	0.236	1.349	0.073^*^	1.027	0.780	1.069	0.625
Jewish		0.592	0.000^***^	0.707	0.015^**^	1.340	0.328	0.555	0.005^***^	0.684	0.153
Unknown		1.637	0.000^***^	0.950	0.757	0.769	0.451	1.037	0.877	0.950	0.870
Social class											
Elite		0.642	0.043^**^	0.731	0.161	0.678	0.518	0.481	0.046^**^	1.220	0.544
Middle class		0.785	0.001^***^	0.868	0.073^*^	0.947	0.761	0.803	0.047^**^	0.956	0.760
Skilled		0.849	0.015^**^	0.821	0.004^***^	0.796	0.154	0.829	0.041^**^	0.824	0.139
Semi-skilled		0.867	0.111	0.831	0.042^**^	0.669	0.077^*^	0.841	0.156	0.929	0.664
Unskilled		(ref)		(ref)		(ref)		(ref)		(ref)	
Unknown		2.075	0.000^***^	0.568	0.000^***^	0.681	0.231	0.546	0.003^***^	0.506	0.036^**^
Single mother		3.371	0.000^***^	4.976	0.000^***^	3.441	0.000^***^	5.643	0.000^***^	5.024	0.000^***^
Season of birth											
Spring		(ref)		(ref)		(ref)		(ref)		(ref)	
Summer		1.027	0.709	0.987	0.852	1.153	0.387	0.874	0.153	1.149	0.325
Autumn		1.088	0.217	0.998	0.980	1.052	0.759	0.824	0.038^**^	1.400	0.011^**^
Winter		1.081	0.248	1.078	0.265	1.215	0.227	0.985	0.866	1.223	0.141
Tax value											
<100		(ref)		(ref)		(ref)		(ref)		(ref)	
100–200		0.874	0.018^**^	1.019	0.770	1.115	0.460	1.007	0.935	0.986	0.906
200+		0.724	0.000^***^	0.946	0.477	1.115	0.549	0.910	0.369	0.921	0.579
Backstreet alley		1.190	0.000^***^	1.120	0.137	0.992	0.966	1.253	0.024^**^	0.926	0.620
Distance to midwife											
<75		(ref)		(ref)		(ref)		(ref)		(ref)	
75–150		0.887	0.068^*^	0.893	0.089^*^	1.033	0.839	0.840	0.053^*^	0.934	0.590
150–300		0.999	0.985	1.016	0.807	1.085	0.599	1.007	0.932	1.018	0.887
300–600		1.173	0.112	1.078	0.516	1.163	0.571	1.074	0.652	1.066	0.771
600+		1.518	0.083^*^	1.210	0.494	2.139	0.202	1.402	0.331	0.452	0.303
Water quality canals											
Seafront		(ref)		(ref)		(ref)		(ref)		(ref)	
‘Best’		1.035	0.818	0.917	0.631	0.784	0.605	0.928	0.756	0.962	0.911
‘Intermediate’		0.972	0.679	1.046	0.566	0.957	0.815	1.165	0.155	0.880	0.378
‘Worst’		1.192	0.006^***^	1.124	0.315	1.208	0.484	1.188	0.277	0.958	0.848
House in front of canal		1.053	0.170	0.914	0.156	0.904	0.495	0.936	0.448	0.899	0.376
Neighbourhood											
Centre		(ref)		(ref)		(ref)		(ref)		(ref)	
Jewish quarter		0.704	0.001^***^	0.847	0.188	1.110	0.713	0.811	0.234	0.780	0.311
Islands		1.045	0.613	1.104	0.342	1.681	0.035^**^	1.027	0.852	0.987	0.948
Jordaan		1.124	0.086^*^	1.008	0.951	1.491	0.181	0.918	0.609	0.971	0.904
Northern canals		0.724	0.005^***^	0.773	0.042^**^	0.913	0.769	0.719	0.057^*^	0.808	0.361
Southern canals		0.959	0.624	0.972	0.807	0.965	0.905	1.054	0.729	0.827	0.405
Plantage Weesper		0.860	0.141	0.934	0.526	0.994	0.983	0.929	0.615	0.925	0.697
Outer areas		1.593	0.001^***^	1.521	0.039^**^	1.631	0.320	1.564	0.097^*^	1.352	0.452

*Note(s)*: Univariate models were run for each variable separately; neonatal period = first 28 days, early post-neonatal = from day 29 up to the 6^th^ month, later post-neonatal = from the 7^th^ up to the 12^th^ month; significance

^*^ *p* < 0.1;

^**^ *p* < 0.01;

^***^ *p* < 0.001; (ref.) = reference category.

*Source(s)*: Own calculations based on linked data from Amsterdam cause-of-death database, Amsterdam birth (1856) and death (1856–57) certificates, Amsterdam population register 1851–53 and HISGIS Amsterdam.

Table 2 also highlights the detrimental effect on infant survival chances of being born to a lone mother. Even though this category includes widows in our dataset, it mainly consists of children born to unmarried women. Other scholars have also noted this higher risk of infant death among children born out of wedlock and commonly attribute it to a lack of care and breastfeeding caused by the need of mothers to go to work due to their unfavourable financial situations.^83^ These adverse effects are mirrored by the reduced survival chances of infants among the ‘unknown’ social class variable, of which 80 per cent consisted of infants born to single mothers.

Religious denomination is another sociocultural characteristic that could affect infant mortality risks. Differentiating between 10 different religions Ekamper and Van Poppel have already demonstrated that differences in HR were relatively modest between most religions in mid-nineteenth-century Amsterdam, with the notable exception of the Jewish population.^84^ Our univariate model confirms this finding for the birth cohort of 1856 and indicates that Jewish infant mortality risks were reduced by almost 40 per cent compared to other religious denominations in the city. Even in our multivariate model, which includes all sociodemographic variables and additional variables such as a street being considered a backstreet alley, distance to midwife, water quality of canals and administrative neighbourhood, infant mortality risks of the Jewish population remained significantly reduced. With the Amsterdam Jewish quarters infamous for their poverty and crowdedness, explanations for this mortality advantage are generally not sought in the socioeconomic sphere. Instead, this reduced mortality risk is commonly attributed to sociocultural characteristics, like more and longer breastfeeding, better hygienic practices, diet and nutrition, as well as a greater willingness to embrace modern medicine.^85^

Whereas certain aspects of the Jewish communities’ lifestyle significantly reduced mortality risks of their infants, socioeconomic characteristics seem to have played a major role in determining the fates of the rest of Amsterdam’s population in the mid-nineteenth century. The impact of social class on infant mortality has been subject to a substantial and continuing international debate centred on questions about whether class differentials can be discerned in different times and places, whether this constituted a social gradient, and even what the direction of such a gradient might have been.^86^ While some studies observed only modest or no effects of occupational status on infant mortality levels, others did find substantial class-mortality differences during the nineteenth century, with lower infant mortality among the upper social classes in England and Wales, Venice, Stockholm, Rostock, Paris and in various regions in the Netherlands.^87^ Measured through the occupational status of the infant’s father or mother, our univariate model shows a clear and largely significant social gradient in all-cause infant mortality. Compared to infants of unskilled workers, the risks of infant mortality decreased by 15 per cent for infants of skilled workers, by 21 per cent for the middle class and by 36 per cent for the city’s elite.

The effect of social class is reduced but does not disappear in the multivariate model. Even though the overt distinctions between semi-skilled, skilled and middle class largely fell away, increased mortality risks among infants born to unskilled labourers persisted when all other sociodemographic and environmental factors were controlled for. Differences in breastfeeding and fertility patterns, quality of food, care, personal hygiene and sanitary conditions between social groups are cited as potential mechanisms through which socioeconomic status might have impacted the survival chances of infants.^88^

In order to further probe this social gradient in infant mortality, we used multi-episode multivariate models (Table 2, models 3–5). Factors affecting mortality risks changed over the first year of life. In the early stages, mortality is still largely determined by foetal development and problems during childbirth, particularly in the first 4 weeks after birth. Afterwards, environmental influences affecting the route of spread of contagious diseases are believed to have increased in importance. We therefore distinguish between the neonatal period, ‘early’ post-neonatal infancy and ‘later’ post-neonatal infancy. This approach reveals that the social gradient we observed in infant mortality was concentrated in the first months of life rather than the later ones. Where mortality hazards increased down the social ladder during early post-neonatal infancy in a statistically significant manner, this effect is less clear for the neonatal period and is even absent among older infants.

Before turning to the cause-specific analyses, we may review the impact of other factors on the social patterning of infant mortality beyond the parents’ occupational status. Given the predominance of social differentiation among early post-neonatal infancy rather than the neonatal period, it is not surprising that the nearest distance to a midwife did not significantly affect infant mortality differentials. We assume that this lack of association is related to the spread of midwives throughout most of the city. Our GIS analysis suggests that three quarters of addresses in the middle of the nineteenth century were less than 200 m removed from the nearest midwife. Midwives also commonly lived in the city’s poorer neighbourhoods. This lack of social segregation, where only particular (wealthier) groups would have a midwife nearby, makes it understandable why distance to a midwife does not appear to have been an important or socially differentiated risk factor for infant mortality in Amsterdam. In general, Dutch women gave birth to their children at home, regardless of their socioeconomic status. If any obstetric practitioner assisted in the delivery at all, it was usually the midwife; obstetric doctors or man-midwives were only called in in difficult or dangerous cases.^89^

The limited role of environmental factors, such as housing conditions, nearby water quality-related conditions, and the location of the neighbourhood, is equally unsurprising in this regard. In general, the effects of intra-urban environmental inequalities in access to clean water and sewerage, as well as housing conditions and overcrowding resulting in increased exposure to disease, are considered to have been stronger in the later stages of infancy.^90^ However, considering the timing of sanitary improvements in Amsterdam, the broadly shared absence of sanitary infrastructures in the middle of the nineteenth century likely precluded a social patterning of mortality for any age group.

The multivariate model does reveal reduced infant mortality risks in the wealthiest neighbourhoods in the northern canal district during early post-neonatal infancy and, conversely, heightened risks in backstreet alley slums above and beyond individual class characteristics. Contemporary public health reports of the early 1860s had already drawn attention to the devastating effects of these slums on general mortality.^91^ However, it is unclear to what extent these variables capture the effects of environmental exposures or reflect differences in social dimensions such as labour participation and maternal health. In their studies on nineteenth-century Rostock and Venice, respectively, Mühlichen and Derosas observed a strong differentiation by social class in neonatal deaths and much less pronounced differences afterwards.^92^ This is largely in line with our results. Their conclusion that these class differentials in early infant mortality risks were probably related to patterns of maternal care and breastfeeding and/or severe deficits in maternal nutritional levels among the lower social classes provides a useful vantage point for a further exploration into the specific causes of death.

## Cause-Specific Mortality

Before delving further into what drove the observed social differences in all-cause mortality, a closer look at the general distribution of the different causes of death of infants in 1856 is imperative. As is shown in Table 3, infectious diseases made up just under a quarter of infant deaths (23 per cent), led by airborne diseases (13 per cent), with a smaller role for water- and foodborne diseases (8 per cent) and other infectious diseases (2 per cent). Scholars relate infectious diseases to exogenous factors such as nutrition, sanitation and hygiene, as well as social, economic and climatic conditions. They typically originate in the post-neonatal period and – due to the prevalence of water- and foodborne diseases in many places – were particularly dominant in the summer season when substitute nutrition perished more easily.^93^ The role of water- and foodborne diseases was substantially lower than the 23 per cent reported in Stockholm between 1878 and 1926, but there is evidence that this causal group would rise in importance in Amsterdam during the second half of the nineteenth century when sanitary conditions worsened.^94^ Until then, more infants in Amsterdam are registered as succumbing to airborne diseases, a group of diseases commonly associated with poor housing and living conditions in cities in particular.^95^

**Table 3.** Infant deaths by cause of death and age at death, Amsterdam, children born in 1856

Cause of death (infant cat)	Neonatal (0–28 days)	Early post-neonatal (29 days to 5 months)	Later post-neonatal (6–11 months)	Total	Per cent
Airborne diseases	8	100	112	220	13.0
Water-food borne diseases	11	74	47	132	7.8
Other infectious diseases	4	14	19	37	2.2
Subtotal infectious diseases	23	188	178	389	22.9
Congenital and birth disorders	42	90	21	153	9.0
Weakness	66	200	63	329	19.4
Convulsions	82	150	57	289	17.1
Teething	0	3	14	17	1.0
External causes	1	0	0	1	0.1
Other causes	18	60	35	113	6.7
Unknown and ill-defined^*^	80	236	88	404	23.8
Total	312	927	456	1695	100.0
Per cent	18.4	54.7	26.9	100.0	

^*^Combined category of ‘stated to be ‘unknown’’ (*N* = 267), known deaths not registered in the cause-of-death registers (*N* = 97) and ‘ill-defined and unknown’(*N* = 31).

*Source(s)*: Own calculations based on linked data from Amsterdam cause-of-death database and Amsterdam death certificates (1856–57).

Several other important disease categories were related to endogenous factors, which are biological factors related to the formation of the foetus in the womb determining an infant’s constitution at birth. Death often occurs within the first 4 weeks of life.^96^ In Amsterdam, just under one-tenth of infant deaths were categorised as congenital and birth disorders, among which an expected proportion of over a quarter was indeed neonatal. More vague categories such as debility and convulsions represented 19 and 17 per cent of infant deaths. ‘Debility,’ ‘weakness’ or ‘wasting’ are generally considered to have endogenous causes, with low birth weight as a risk factor and influenced by early gestation as well as higher maternal age at childbirth, poor harvest yields, birth rank and birth spacing.^97^ The category of convulsions is more speculative, as no consensus has been reached about the underlying causes.^98^ Nineteenth-century medical literature attributed ‘eklampsie’ to a wide range of causes, from a cold and pneumonia to whooping cough and disorders in the digestive system. Today’s scholars are equally divided on the subject, with convulsions being classified as ‘non-infectious disease and accidents’ for Stockholm, as ‘other infectious diseases’ for various regions in the Netherlands, and as ‘water- and foodborne infectious diseases’ for Maastricht and Tartu because of a notable summer peak.^99^ With convulsions lacking such a distinct summer peak in nineteenth-century Amsterdam, it remains somewhat unclear how these convulsions should be understood.

A final notable cause-of-death category in Amsterdam in 1856 was that of ‘unknown and ill-defined’ (24 per cent). As the name indicates, the causes in this category were either ill-defined and were absent from the cause-of-death registers or, in the majority of cases, merely listed as ‘unknown.’ According to Janssens and Riswick, the importance of this group would gradually diminish and even completely disappear from Amsterdam’s cause-of-death registers before the 1880s.^100^ They relate this development to the New Public Health Inspectorate Act and the Medical Practitioners Act of 1865, through which the Dutch government made it mandatory for medical doctors throughout the country to provide a medical certificate with the cause of death before the issuing of a death certificate.^101^ As such, they suggest that the decline of the ‘unknown’ category may well represent changing diagnostic practices, both in terms of increases in the incidence of medical attendance and changes in the practices used to register cause-of-death diagnoses. Other vague categories such as ‘debility’ disappeared in roughly the same period, and the use of ‘convulsions’ as a cause of death also declined significantly during the second half of the nineteenth century. As such, the substantial ‘unknown’ category may well represent the deaths of infants who, for one reason or another, largely escaped the attention and consideration of the medical community at this point in time.

Which cause-of-death categories drove the observed social differentiation in infant mortality can be probed with a competing risk analysis. Results with respect to the social class variable are shown in Table 4 (and visualised in Supplementary Figure S3).^102^ An investigation of the different causes reveals a lack of social class differentials for convulsions and weakness; no clear pattern emerges among the infants belonging to the different social classes for these causal groups. None of the distinguished infectious disease groups contributed to the aforementioned social differentiation either. Due to their similarities, water- and foodborne, airborne and other infectious diseases are presented in the models as one category. Whether analysed separately or together, infants who died from infectious diseases revealed no clear socially differentiated pattern in the mid-nineteenth century. This outcome is not unexpected, seeing as there is little social differentiation in the post-neonatal period.

**Table 4.** Social class subdistribution hazard ratios for infant mortality by age at death and cause of death, Amsterdam, children born in 1856

Social class (SOCPO)	Total	Neonatal	Early post-neonatal	Later post-neonatal	Total	Neonatal	Early post-neonatal	Later post-neonatal
	All causes				Infectious diseases			
Elite	0.731	0.678	0.481^**^	1.220	1.121	-	0.527	1.763
Middle class	0.868^*^	0.947	0.803^**^	0.956	0.778^*^	1.406	0.799	0.623^**^
Skilled workers	0.821^***^	0.796	0.829^**^	0.824	0.782^*^	0.647	0.886	0.622^**^
Semi-skilled workers	0.831^**^	0.669^*^	0.841	0.929	0.618^**^	-	0.565^*^	0.657
Unskilled workers (ref.)	1.000	1.000	1.000	1.000	1.000	1.000	1.000	1.000
Unknown	0.568^***^	0.681	0.546^***^	0.506^**^	0.554^*^	1.246	0.703	0.325^**^
	Congenital and birth disorders				Weakness			
Elite	0.308	0.674	-	-	0.595	1.237	0.660	-
Middle class	0.390^***^	0.382^*^	0.436^*^	0.248	1.177	1.353	1.033	1.364
Skilled workers	0.654^**^	0.320^**^	0.687	1.347	0.953	1.049	0.810	1.132
Semi-skilled workers	0.594^*^	0.198^**^	0.810	1.386	0.839	0.545	0.966	0.557
Unskilled workers (ref.)	1.000	1.000	1.000	1.000	1.000	1.000	1.000	1.000
Unknown	0.919	0.996	0.618	2.637	0.746	1.376	0.653	0.278^*^
	Convulsions				Unknown and ill-defined causes			
Elite	0.917	0.902	0.347	1.942	0.415	-	0.329	0.784
Middle class	1.079	0.708	0.864	2.685^**^	0.696^**^	0.934	0.654^*^	0.878
Skilled workers	1.074	1.200	0.932	1.000	0.821	0.981	0.823	0.743
Semi-skilled workers	1.061	0.928	0.931	1.805	1.061	1.447	0.991	1.036
Unskilled workers (ref.)	1.000	1.000	1.000	1.000	1.000	1.000	1.000	1.000
Unknown	1.010	0.675	1.020	1.449	0.294^***^	0.344^***^	0.352^***^	0.201

*Note(s)*: Neonatal = first 28 days, early post-neonatal = from day 29 up to the 6^th^ month, later post-neonatal = from the 7^th^ up to the 12^th^ month; significance ^*^p < 0.1; ^**^p < 0.01; ^***^p < 0.001; (ref.) = reference category; - = omitted variable categories; for the full multivariate Fine‐Grey competing risk subdistribution hazard model estimations see the Supplementary Tables S1 to S5; Cox all-causes model estimates (Table 1, models 2–5) added for comparison reasons.

*Source(s)*: Own calculations based on linked data from Amsterdam cause-of-death database, Amsterdam birth (1856) and death (1856–57) certificates, Amsterdam population register 1851–53 and HISGIS Amsterdam.

By comparison, the causal groups of ‘congenital and birth disorders’ as well as ‘unknown and ill-defined’ causes of death do indicate a significant advantage for skilled workers and the middle class compared to the unskilled class, with a similar point estimate direction for the elite in survival chances. As previously indicated by the all-cause Cox models, it is in the neonatal period and early post-neonatal infancy that the mortality risks decrease up the social ladder. During the first 28 days of life, chances of dying from congenital or birth disorders were more than 60 per cent lower among infants belonging to Amsterdam’s middle class than among the city’s unskilled workers; during early infancy risks, remained 50 per cent lower. As ‘prenatal diseases,’ congenital and birth disorders are commonly viewed in relation to the health of the mother.^103^ The substantial social differences can therefore be considered a reflection of a social gradient of maternal health across the city.

For the unknown and ill-defined causes, overall infant mortality risks were over 30 per cent lower for middle-class inhabitants than for unskilled labourers. Even though the social differentiation among unknown causes of death is less significant than deaths due to congenital and birth disorders, the effect directions in especially early post-neonatal infancy are similar to the ones from congenital and birth disorders and also quite distinct from those found for other causal groups such as infectious diseases. Additionally, it is noteworthy that the waning of unknown causes of death due to the professionalisation of diagnostic practices during the 1860s and 1870s was accompanied by a growing share of congenital and birth disorders.^104^ This simultaneous rise and fall of these two causal groups may be an indication that at least a substantial portion of the unknown and ill-defined causes of death should be counted towards the congenital and birth disorders. That this was not the case before new legislation required certification for all deaths may furthermore suggest that many of those recorded as having an ‘unknown’ cause of death belonged to a subsection of society less well served by medical doctors.^105^

## Unknown Causes of Death

We employ two different methods to verify whether the ‘unknowns’ may be likened to ‘congenital and birth disorders’ in mid-nineteenth-century Amsterdam. First, we compare the age distributions of the different causal groups. Congenital and birth disorders are associated with early death: deaths in the first week (perinatal or early neonatal mortality) and within the first 28 days of life (neonatal mortality) often occurred among babies with very particular health problems related to premature births, low birth weights and congenital malformations.^106^ The size of our dataset precludes a separate analysis of perinatal mortality, but a sizeable portion (27 per cent) of the congenital and birth disorder category did occur in the first month. For the unknown causes of death, this was true for 20 per cent, and for debility, 20 per cent was neonatal.

A more thorough statistical test of the hypothesis that the two samples are drawn from the same population based on age distribution is provided by the Epps-Singelton test. This is a non-parametric test (meaning it does not assume anything about the underlying distribution) and, contrary to the widely used Kolmogorov-Smirnov test, is also valid for discrete (non-continuous) outcomes like our survival time measured in days.^107^Table 5 summarises the results from the Epps-Singleton test for various relevant (combinations of) causal groups. The null hypothesis is that the two samples are taken from populations with equal distribution; this null hypothesis is rejected when there is less than a 5 per cent chance of the sample result if the null hypothesis were true. In terms of the age distribution of our cause-of-death groups, this means that the unknown causes are considered statistically dissimilar from infectious diseases, from convulsions and from the total of causes not categorised as unknown. On the other hand, it is considered statistically likely that the deaths due to unknown causes are drawn from the same population as those dying from debility as well as from congenital and birth disorders. When paired together, the distributions of survival time between these combined causal groups and the remaining other causes of death differ in a significant way. In terms of age distribution, the unknown causes of death are commensurate with congenital and birth disorders as well as debility.

**Table 5.** Outcomes of the Epps-Singleton test on age distributions of different cause-of-death groups, with the null hypothesis that the distributions are identical

Sample 1	Sample 2	*p*-value
Unknown	All other	0.000
Unknown	Infectious diseases	0.000
Unknown	Convulsions	0.019
Unknown	Congenital and birth disorders	0.148
Unknown	Debility	0.593
Unknown and congenital and birth disorders	All other	0.000
Unknown and congenital and birth disorders and debility	All other	0.000

*Source(s)*: Own calculations based on data from Amsterdam cause-of-death database.

A second way to better understand the unknown causes of death is to compare the effects of various variables on mortality risks between different causal groups in the competing risk models (Supplementary Tables S1 to S5). Such a comparison partially confirms our previous findings: the aetiological mechanisms that characterise the unknown causes seem congruent with those for congenital and birth disorders but lack resemblance to the other cause-of-death groups. No clear and linear social differentiation can be observed for those dying from convulsions, infectious diseases or debility. All of these three groups lack the unambiguous socioeconomic mortality disadvantages that we found for all-cause infant mortality, whether measured through social class, property tax value or a backstreet alley location. Living in a house in front of a canal also significantly reduced chances of dying from convulsions during late infancy, though it is unclear whether this is related to better access to fresh water sold from boats via the canals or actually represents relative status advantages associated with living along Amsterdam’s canals.^108^ Finally, the neighbourhood variable does not really seem to capture as many detrimental ‘neighbourhood effects’ on infant mortality risks in the lower-class Jordaan and the Islands neighbourhoods as one might expect. However, the models do underscore infants’ reduced mortality risks in the Jewish neighbourhood as well as in the wealthier northern canal belt.^109^

Socioeconomic predictors seem to have played pivotal roles in infant mortality risks for both congenital and birth disorders and unknown causes of death. We cannot consider the two causal groups as being identical based on the outcomes of the competing risk models since the strength and even direction of certain variables diverge. For example, Catholic infants experienced significantly higher risks of dying from congenital and birth disorders in the neonatal period according to our data, but no such effect can be discerned for the unknown causes of death. Nevertheless, both causal groups displayed stronger social class-mortality advantages up the social ladder in the neonatal period as well as post-neonatal infancy than other causes of death. Other socioeconomic variables also came up as significant, though in varied forms: risks of dying from congenital and birth disorders were higher in backstreet alley dwellings during early and later post-neonatal infancy, while property tax value was not significant. For the unknown causes of death, the backstreet alley variable has the same effect during early post-neonatal infancy, but here the higher property tax values did also reduce infants’ mortality risks. By itself, the comparison of effects of different variables on the various disease categories does not provide a clear-cut answer to the question whether the unknown causes of death can be conclusively equated with congenital and birth disorders. Combined with the evidence from the distribution test, however, we can cautiously surmise that these two causal groups can be viewed in conjunction with each other, driven as they were by similar socioeconomic forces largely absent in the other causal groups.

The social differentiation in mortality due to congenital and birth disorders and unknown causes brings up questions about intra-urban disparities in health or care as well as access to medical provisions. Generally speaking, survival chances of infants are said to have been more dependent on their mother’s constitution and feeding practices during the first half year of their life than on the economy of the family as a whole.^110^ A mother’s nutritional state during pregnancy would primarily impact infant deaths through immaturity of the foetus and congenital effects. With rather vague disease terms as ‘paedatrophia’ and ‘atrophia infantum,’ most infants in the category of congenital and birth disorders were probably already weak from birth, with low birth weight.^111^ This disadvantage was heightened if mothers were also of frail health and unable to breastfeed an infant. The impact of breastfeeding, or more specifically, the risks accompanying the use of artificial foods given the unhygienic circumstances of the time, have been well documented.^112^ Stark social differences across mid-nineteenth-century Amsterdam are conceivable, not only because maternal health was itself strongly affected by real income levels but also because of differences in female employment across the social spectrum and the necessity for poorer women to return to work more quickly.^113^ Paid female employment is primarily believed to have impacted infant mortality through the reduced amount and duration of breastfeeding – though there are indications that additional home help with child care could offset this relationship – and by reducing the mother’s overall health status.^114^ In Amsterdam, many women engaged in paid labour: at the very least a quarter of the potential labour force consisted of women according to the 1,859 labour force count, which was likely subject to a large degree of under registration.^115^ Unfortunately, these important dimensions cannot readily be measured in a more specific way beyond our proxies, like social class. Nevertheless, infants’ significantly reduced survival chances due to unknown causes among the lower working-class Eastern and Western Island neighbourhoods (neighbourhood 3 on Supplementary Figure S1), and their much lower mortality risks in the wealthiest neighbourhoods in the northern canal belt (neighbourhood 5), remain intriguing nudges in that direction.

In addition to inequalities in maternal health across the city, disparities in access to medical doctors could also help shape social differences in the recording of infant mortality. Earlier research already indicated that distances to medical practitioners other than midwives did not significantly impact infant mortality in mid-nineteenth-century Amsterdam.^116^ And in line with our all-cause mortality findings, our cause-specific models are also unable to discern any clear-cut or notable effects of the proximity of midwives on the infant causes of death either.

It does seem plausible that differential certification practices resulted in an unequal distribution of unknown causes of death based on social class, unrelated to proximity. The previously mentioned concurrent rise and fall of congenital and birth disorders and unknown causes due to increased professionalisation of diagnostic and medical certification practices points to a credible connection between these causal groups. The medical doctor Israëls already noted the high numbers of unknown causes of death among infants during the 1850s in some of Amsterdam’s poorest neighbourhoods, such as the Eastern Islands and Jordaan neighbourhoods. He attributed them to ‘neglect, lack of civilisation and misery’ in these parts, but also noted that poor medical supervision may be to blame.^117^ In the context of such disparities, Israëls put forward the idea that the city might be facing a medico-political problem: were doctors not consulted enough among certain parts of society or did medical practitioners not take their work there seriously enough or perhaps a combination of both? For nineteenth-century Scotland, Garrett and Reid have convincingly argued that rural areas and poorer places and people were likely less well served by medical practitioners.^118^ They suggested that a significant number of unknown causes of death should be taken as an indication of low levels of medical certification and/or effective medical provision. Luque de Haro et al. have made a comparable claim for ill-defined diseases in late eighteenth and early nineteenth-century Spain.^119^

We propose that similar mechanisms were at play on an intra-urban level in mid-nineteenth-century Amsterdam. While we have found no evidence that ineffective medical provisions *caused* infant deaths, we suggest that socially differentiated (in)attention impacted the likelihood of a specific cause of death being *identified*. Combined with the inference that the likeness of ‘unknown and ill-defined’ deaths to deaths due to ‘congenital and birth disorders’ is suggestive of stark maternal health disparities across the city, our scrutiny reveals a world of difference, with unequal mortality hazards for mother and child structured along the same class lines.

## Conclusion

This paper analysed inequalities in infant mortality in Amsterdam in 1856. It took as a starting point the important discussion about the long-term evolution of social inequalities in mortality, which commonly presupposes that the majority of deaths were unavoidable and that mortality was largely invariant between social groups prior to the epidemiological transition.^120^ With high and fluctuating levels of mortality due to infectious diseases, low life expectancy and high levels of infant mortality, it would still take the city of Amsterdam some 20 years until it would pass from what Omran called the ‘Age of Pestilence and Famine’ into the ‘Age of Receding Pandemics.’^121^ Applying different methods of survival analysis to novel individual-level cause-of-death data, this paper sought to answer whether the burden of infant death was indeed distributed equally across Amsterdam’s population in 1856 and, if not, what the disease categories might be able to tell us about the mechanisms underlying these disparities.

Our survival analyses have brought to light important social differences in infants’ mortality risks in mid-nineteenth-century Amsterdam. Several of these have also been observed in other studies, such as the overall mortality disadvantage for male infants, for multiple births and for births to an older or single mother, as well as the advantageous position of Jewish infants compared to other religious denominations.^122^ We observed an overt and largely significant social differentiation in the univariate model of all-cause infant mortality, with children born to middle-class parents experiencing a mortality risk that was 22 per cent lower than that experienced by the children of unskilled workers. The mortality disadvantage of unskilled labourers was maintained in the multivariate models, especially in early post-neonatal infancy (1–5 months), although differences between the semi-skilled, skilled and middle class seem to have been minimal when all other sociodemographic and environmental variables are controlled for.

The existence of these health inequalities raises important questions about the societal mechanisms that have produced them. The cause-specific analysis reveals that the disease groups responsible for the social differentiation in all-cause mortality were congenital and birth disorders as well as unknown and ill-defined causes of death, the latter of which we argue may be partially counted towards the first category. Substantial improvements in the quality of prenatal and obstetric medicine are assumed to have only occurred from the turn of the twentieth century onwards.^123^ This suggests that the differences in risks of dying from these types of diseases before that time were more likely related to disparities between mothers’ nutritional status and health rather than differential access to health interventions. The social differences in infant mortality likely reflect stark disparities in maternal health across social groups in mid-nineteenth-century Amsterdam.^124^ Whether these deaths can be viewed as preventable is ultimately a philosophical question. Social differentials within the group of infants dying from unknown and ill-defined causes furthermore suggest that medical interest in the recording of causes of death may well have been structured along the same socioeconomic lines.

Comparisons across space but especially over time will help us to interpret the broader meaning of these findings about social inequalities in infant mortality prior to or during the early phase of the epidemiological transition. Time constraints as well as the availability of data necessitated us to limit ourselves to the study of infants born in one single year. Although the 1856 IMR seems to fit broadly with infant mortality trends around that period, further inquiry into multiple-year averages during should answer questions about its normalcy and representativeness. A comparison with later time periods could also provide insight into the trajectories of the different disease categories during the epidemiological transition. Clouston et al.’s model about the production, reduction and eradication of mortality inequalities may provide a useful vantage point for such comparisons. Was the social differentiation in birth and congenital disorders maintained until the decline of infant mortality rates during the 1880s or did maternal health improve before that? What effect did advances in prenatal and obstetric care after the turn of the twentieth century have, and in a broader sense, to what extent do Clouston et al.’s ideas about the mechanisms of inequality align with historical reality? Additionally, when did social inequalities in infectious diseases increase or reduce, and which mechanisms were responsible? And to what extent are these patterns maintained for child mortality?

Another important addition to future studies could be the incorporation of more extended spatial techniques. It would be valuable to use a nearest-neighbour approach on the population registers to empirically assess the impact of one’s social surroundings instead of relying on (groupings of) administrative neighbourhoods. Nevertheless, it is significant that inequality during the first year of life existed in Amsterdam decades before the decline of infant mortality set in. It brings into view the long run-up to monumental historical change and demonstrates the importance of both age- and cause-of-death-specific data for its fuller comprehension.

## Supplementary Material

Supplementary material mentioned in the text is available at www.shm.oxfordjournals.org.

hkae066_suppl_Supplementary_Material

## Footnotes

1 J. C. Riley, *Rising Life Expectancy: A Global History* (Cambridge: Cambridge University Press, 2001), 1, https://doi.org/10.1017/CBO9781316036495; J. P. Mackenbach, *A History of Population Health: Rise and Fall of Disease in Europe* (Leiden: Brill, 2020), 2, https://brill.com/view/title/57111.

2 F. W. A. van Poppel, ‘Het komen en gaan van ziekten’, in H. F. P. Hillen, E. S. Houwaart and F. G. Huisman, eds, *Medische geschiedenis* (Houten: Bohn Stafleu van Loghum, 2018), 15.

3 T. Bengtsson and F. W. A. van Poppel, ‘Socioeconomic Inequalities in Death from Past to Present: An Introduction’, *Explorations in Economic History*, July 2011, 48, 343–56, https://doi.org/10.1016/j.eeh.2011.05.004.

4 Ó. Garðarsdóttir, ‘Saving the Child: Regional, Cultural and Social Aspects of the Infant Mortality Decline in Iceland, 1770–1920’ (Doctoral Thesis, Umeå, Umeå University, 2002), 32–33, https://urn.kb.se/resolve?urn=urn:nbn:se:umu:diva-56811; J. Knodel, ‘Two Centuries of Infant, Child and Maternal Mortality in German Village Populations’, in A. Brändström and L. Tedebrand, eds, *Society, Health and Population during the Demographic Transition* (Stockholm: Almqvist and Wiksell International, 1988), 23–48; W. R. Lee and P. Marschalck, ‘Infant Mortality in Bremen in the 19th Century’, *The History of the Family*, 2002, 7, 557–83, https://doi.org/10.1016/S1081-602X(02)00127-6; C. Rollet, ‘Allaitement, mise en nourrice et mortalité infantile en France a la fin du XIXe siècle’, *Population (French Edition)*, 1978, 33, 1189–203, https://doi.org/10.2307/1531965; R. Woods, Naomi Williams, and Chris Galley, ‘Differential Mortality Patterns among Infants and Other Young Children: The Experience of England and Wales in the Nineteenth Century’, in C. A. Corsini and P. P. Viazzo, eds, *The Decline of Infant and Child Mortality* (Leiden: Brill, 1997), https://doi.org/10.1163/9789004638679_006; P. Razzell and C. Spence, ‘Poverty or Disease Environment? The History of Mortality in Britain, 1500–1983’, in M. Breschi and L. Pozzi, eds, *The Determinants of Infant and Child Mortality in Past European Populations* (Udine: Forum, 2004), 43–66; H. Jaadla et al., ‘Infant and Child Mortality by Socio‐economic Status in Early Nineteenth‐century England’, *The Economic History Review*, November 2020, 73, 991–1022, https://doi.org/10.1111/ehr.12971; M. Dribe and O. Karlsson, ‘Inequality in Early Life: Social Class Differences in Childhood Mortality in Southern Sweden, 1815–1967’, *The Economic History Review*, 2002, 75, 475–502, https://doi.org/10.1111/ehr.13089.

5 A. Perrenoud, ‘L’inégalité sociale devant la mort a Genève au XVIIe siècle’, *Population*, 1975, 30, 221–43, https://doi.org/10.2307/1530652; R. Schumacher and M. Oris, ‘Long-Term Changes in Social Mortality Differentials, Geneva, 1625–2004’, *Explorations in Economic History, Socioeconomic Inequalities in Death*, 1 July 2011, 48, 357–65, https://doi.org/10.1016/j.eeh.2011.05.011; A. Fornasin, M. Breschi, and M. Manfredini, ‘Environment, Housing, and Infant Mortality: Udine, 1807–1815’, in D. Ramiro Fariñas and M. Oris, eds, *New Approaches to Death in Cities during the Health Transition* (Cham: Springer International Publishing, 2016), 43–54, https://doi.org/10.1007/978-3-319-43002-7_3; M. Breschi et al., ‘Socioeconomic Conditions, Health and Mortality from Birth to Adulthood, Alghero 1866–1925’, *Explorations in Economic History*, 2011, 48, 366–75, https://doi.org/10.1016/j.eeh.2011.05.006; R. Derosas, ‘Socio-Economic Factors in Infant and Child Mortality: Venice in Mid-Nineteenth Century’, in M. Breschi and L. Pozzi, eds, *The Determinants of Infant and Child Mortality in Past European Populations* (Udine: Forum, 2004), 105–22; J. P. Ferrie, ‘The Rich and the Dead: Socioeconomic Status and Mortality in the U.S., 1850–1860’, in D. L. Costa, ed, *Health and Labor Force Participation Over the Life Cycle: Evidence from the Past* (Chicago: University of Chicago Press, 2003), 11–50; N. Williams, ‘Death in Its Season: Class, Environment and the Mortality of Infants in Nineteenth-Century Sheffield’, *Social History of Medicine*, 1992, 5, 71–94, https://doi.org/10.1093/shm/5.1.71.

6 A. Janssens and I. Devos, ‘The Limits and Possibilities of Cause of Death Categorisation for Understanding Late Nineteenth Century Mortality’, *Social History of Medicine*, 30 August 2022, hkac040, https://doi.org/10.1093/shm/hkac040; M. Murkens, B. Pelzer, and A. Janssens, ‘Transitory Inequalities: How Individual-Level Cause-Specific Death Data Can Unravel Socioeconomic Inequalities in Infant Mortality in Maastricht, the Netherlands, 1864–1955’, *The History of the Family*, 2022, 1–36, https://doi.org/10.1080/1081602X.2022.2084442; S. A. P. Clouston et al., ‘A Social History of Disease: Contextualizing the Rise and Fall of Social Inequalities in Cause-Specific Mortality’, *Demography*, 2016, 53, 1631–56, https://doi.org/10.1007/s13524-016-0495-5; J. Molitoris, ‘Disparities in Death: Inequality in Cause-Specific Infant and Child Mortality in Stockholm, 1878‒1926’, *Demographic Research*, 2017, 36, 455–500, https://doi.org/10.4054/DemRes.2017.36.15; M. Mühlichen, R. D. Scholz, and G. Doblhammer, ‘Social Differences in Infant Mortality in 19th Century Rostock. A Demographic Analysis Based on Church Records’, *Comparative Population Studies*, 2015, 40, https://doi.org/10.12765/CPoS-2015-03.

7 E. C. Walhout, ‘An Infants’ Graveyard?: Region, Religion, and Infant Mortality in North Brabant, 1840–1940’ (Doctoral Thesis, Tilburg University, 2019), 176.

8 For a recent overview, see A. Reid et al., ‘Fatal Places? Contextual Effects on Infant and Child Mortality in Early Twentieth Century England and Wales’, *Social Science History*, 2023, 47, 400.

9 Mühlichen, Scholz, and Doblhammer, ‘Social Differences in Infant Mortality’, 195.

10 I. Devos and A. Janssens, ‘Introduction’, *TSEG - The Low Countries Journal of Social and Economic History*, 2017, 14, 15, https://doi.org/10.18352/tseg.1001.

11 J. de Vries and A. van der Woude, *The First Modern Economy: Success, Failure, and Perseverance of the Dutch Economy, 1500–1815* (Cambridge: Cambridge University Press, 1997), https://doi.org/10.1017/CBO9780511666841.

12 A. Janssens, ‘The Retreat of Death: Understanding the Rise in Life Expectancy during the First Demographic Transition’, in J. van Bavel et al., eds, *Neurotic Doubt and Sacred Curiosity. Essays in Honour of Koen Matthijs* (Leuven: Centre for Sociological Research KU Leuven, 2021), 437–47.

13 See the special issue edited by Bengtsson and van Poppel, ‘Socioeconomic Inequalities in Death.’

14 A. Antonovsky, ‘Social Class, Life Expectancy and Overall Mortality’, *The Milbank Memorial Fund Quarterly*, 1967, 45, 31–73, https://doi.org/10.2307/3348839.

15 B. G. Link and J. Phelan, ‘Social Conditions As Fundamental Causes of Disease’, *Journal of Health and Social Behavior*, 1995, 80–94, https://doi.org/10.2307/2626958; J. Freese and K. Lutfey, ‘Fundamental Causality: Challenges of an Animating Concept for Medical Sociology’, in Bernice A. Pescosolido et al., eds, *Handbook of the Sociology of Health, Illness, and Healing. Handbooks of Sociology and Social Research* (New York, NY: Springer New York, 2011), 67–81, https://doi.org/10.1007/978-1-4419-7261-3_4.

16 S. A. P. Clouston and B. G. Link, ‘A Retrospective on Fundamental Cause Theory: State of the Literature and Goals for the Future’, *Annual Review of Sociology*, 2021, 47, 140–41, https://doi.org/10.1146/annurev-soc-090320-094912; Clouston et al., ‘A Social History of Disease.’

17 T. Bengtsson and M. Dribe, ‘The Late Emergence of Socioeconomic Mortality Differentials: A Micro-Level Study of Adult Mortality in Southern Sweden 1815–1968’, *Explorations in Economic History, Socioeconomic Inequalities in Death*, 2011, 48, 389–400, https://doi.org/10.1016/j.eeh.2011.05.005; Bengtsson and van Poppel, ‘Socioeconomic Inequalities in Death’, 346.

18 Bengtsson and Dribe, ‘The Late Emergence of Socioeconomic Mortality Differentials’; T. Bengtsson and M. Dribe, ‘Quantifying the Family Frailty Effect in Infant and Child Mortality by Using Median Hazard Ratio (MHR): The Case of Rural Sweden, 1766–1895’, *Historical Methods: A Journal of Quantitative and Interdisciplinary History*, 2010, 43, 15–27, https://doi.org/10.1080/01615440903270299; Dribe and Karlsson, ‘Inequality in Early Life’, 491.

19 Knodel, ‘Two Centuries’; Lee and Marschalck, ‘Infant Mortality in Bremen’; Rollet, ‘Allaitement, mise en nourrice’; Woods, Williams, and Galley, ‘Differential Mortality Patterns’; Razzell and Spence, ‘Poverty or Disease Environment’; Jaadla et al., ‘Infant and Child Mortality by Socio-Economic Status’; Dribe and Karlsson, ‘Inequality in Early Life.’

20 Perrenoud, ‘L’inégalité sociale’; Schumacher and Oris, ‘Long-Term Changes in Social Mortality Differentials’; Fornasin, Breschi, and Manfredini, ‘Environment, Housing, and Infant Mortality’; Breschi et al., ‘Socioeconomic Conditions, Health and Mortality’; Derosas, ‘Socio-Economic Factors’; Ferrie, ‘The Rich and the Dead’; Williams, ‘Death in Its Season’; M. Mühlichen, ‘Premature Mortality in the German Baltic Sea Region Now and Then: Patterns and Trends from a Regional and Historical Perspective’ (Dissertation, Universität Rostock, 2020), 64, https://doi.org/10.18453/rosdok_id00002710.

21 Mühlichen, ‘Premature Mortality’, 64–65.

22 Calculated from Derosas, ‘Socio-Economic Factors’, 114.

23 Dribe and Karlsson, ‘Inequality in Early Life’, 497.

24 Derosas, ‘Socio-Economic Factors’, 119.

25 W. H. Mosley and L. C. Chen, ‘An Analytical Framework for the Study of Child Survival in Developing Countries’, *Population and Development Review*, 1984, 10, 25–45, https://doi.org/10.2307/2807954.

26 Mosley and Chen, 27.

27 Clouston et al., ‘A Social History of Disease’, 1635–37.

28 Clouston et al., 1635, 1639.

29 Murkens, Pelzer, and Janssens, ‘Transitory Inequalities’, 5–6.

30 B. Harris and J. Helgertz, ‘Urban Sanitation and the Decline of Mortality’, *The History of the Family*, 2019, 24, 207–26, https://doi.org/10.1080/1081602X.2019.1605923.

31 Clouston et al., ‘A Social History of Disease’; Clouston and Link, ‘A Retrospective on Fundamental Cause Theory.’

32 J. A. Verdoorn, *Het gezondheidswezen te Amsterdam in de 19e eeuw* (Utrecht: Uitgeverij Het Spectrum, 1965), 102–15; A. Janssens and T. Riswick, ‘What Was Killing Babies in Amsterdam? A Study of Infant Mortality Patterns Using Individual-Level Cause of Death Data, 1856–1904’, *Historical Life Course Studies*, 2023, 13, 243–45, https://doi.org/10.51964/hlcs13438; Murkens, Pelzer, and Janssens, ‘Transitory Inequalities’, 6.

33 Mühlichen, Scholz, and Doblhammer, ‘Social Differences in Infant Mortality’, 195; W. P. Ward, ‘Perinatal Mortality in Utrecht, The Netherlands, 1880–1940’, *Economics & Human Biology*, 2003, 1, 379–98, https://doi.org/10.1016/S1570-677X(03)00070-4.

34 Clouston et al., ‘A Social History of Disease’; Janssens and Devos, ‘The Limits and Possibilities.’

35 Devos and Janssens, ‘Introduction’, 15.

36 A. Janssens, ‘Constructing SHiP and an International Historical Coding System for Causes of Death’, *Historical Life Course Studies*, 2021, 10, 64–70, https://doi.org/10.51964/hlcs9569.

37 M. H. D. van Leeuwen and J. E. Oeppen, ‘Reconstructing the Demographic Regime of Amsterdam 1681–1920’, *Economic and Social History in the Netherlands*, 1993, 5, 87; Verdoorn, *Het gezondheidswezen*, 27.

38 A. Knotter, *Economische transformatie en stedelijke arbeidsmarkt: Amsterdam in de tweede helft van de negentiende eeuw* (Zwolle: Waanders, 1991), 238; J. L. van Zanden, *De industrialisatie in Amsterdam 1825–1914* (Bergen: Octavo, 1987), 78–79.

39 R. Aerts and P. de Rooy, ‘Hoofdstad in aanbouw 1813–1900’, in R. Aerts and P. de Rooy, eds, *Geschiedenis van Amsterdam: Hoofdstad in aanbouw 1813–1900*, vol. III (Amsterdam: SUN, 2006), 13; I. Jager, *Hoofdstad in gebreke: Manoeuvreren met publieke werken in Amsterdam 1851–1901* (Rotterdam: Uitgeverij 010, 2002), 26–27.

40 P. Ekamper and F. W. A. van Poppel, ‘Infant Mortality in Mid-19th Century Amsterdam: Religion, Social Class, and Space’, *Population, Space and Place*, 2019, 25, 3, https://doi.org/10.1002/psp.2232.

41 L. J. Egeling, ‘Boekaankondiging’, *Nederlands tijdschrift voor geneeskunde*, 1861, 5, 139–44; L. J. Egeling, ‘Bijdrage tot de kennis van den gezondheidstoestand der stad Amsterdam in de jaren 1856–1860’, *Nederlands tijdschrift voor geneeskunde*, 1863, 7, 577–92; I. Teixeira de Mattos, *Verslag omtrent den ziektetoestand der stad Amsterdam in 1862 in verband met de Geneeskundige Armendienst* (Amsterdam: Stads-Drukkerij, 1865); J. E. van der Pek and J. Kruseman, *Verslag van een onderzoek naar den toestand der arbeiderswoningen te Amsterdam (oude stad) gehouden door de afdeeling Amsterdam van den Volksbond* (Amsterdam: J. H. de Bussy, 1893).

42 C. Schade, *Woningbouw voor arbeiders in het 19de-eeuwse Amsterdam* (Amsterdam: Van Gennep, 1981).

43 Mühlichen, ‘Premature Mortality’, 34.

44 Ekamper and van Poppel, ‘Infant Mortality’, 3.

45 T. Riswick, S. Muurling, and K. Buzasi, ‘Exploring the Mortality Advantage of Jewish Neighbourhoods in Mid-19th Century Amsterdam’, *Demographic Research* 2022, 46, 723–36, https://doi.org/10.4054/DemRes.2022.46.25; Verdoorn, *Het gezondheidswezen*, 62–68; Ekamper and van Poppel, ‘Infant Mortality’, 3; Lara V. Marks, *Model Mothers: Jewish Mothers and Maternity Provision in East London 1870–1939, Oxford Historical Monographs* (Oxford: Oxford University Press, 1994), 2–3.

46 F. W. A. van Poppel and C. van der Heijden, ‘The Effects of Water Supply on Infant and Childhood Mortality: A Review of Historical Evidence’, *Health Transition Review*, October 1997, 7, 113–48; H. Jaadla and A. Puur, ‘The Impact of Water Supply and Sanitation on Infant Mortality: Individual-Level Evidence from Tartu, Estonia, 1897–1900’, *Population Studies*, 3 May 2016, 70, 163–79, https://doi.org/10.1080/00324728.2016.1176237; L. Kesztenbaum and J. L. Rosenthal, ‘Sewers’ Diffusion and the Decline of Mortality: The Case of Paris, 1880–1914’, *Journal of Urban Economics*, March 2017, 98, 174–86, https://doi.org/10.1016/j.jue.2016.03.001.

47 Verdoorn, *Het gezondheidswezen*, 208–10.

48 Ekamper and van Poppel, ‘Infant Mortality’, 4.

49 Verdoorn, *Het gezondheidswezen*, 211–14.

50 Verdoorn, 225.

51 Verdoorn, 196.

52 R. I. Woods, A. Lokke, and F. van Poppel, ‘Two Hundred Years of Evidence-Based Perinatal Care: Late-Fetal Mortality in the Past’, *Archives of Disease in Childhood - Fetal and Neonatal Edition*, 2006, 91, F445–47, https://doi.org/10.1136/adc.2006.098178.

53 M. J. van Lieburg and H. Marland, ‘Midwife Regulation, Education, and Practice in The Netherlands during the Nineteenth Century’, *Medical History*, July 1989, 33, 296–317, https://doi.org/10.1017/S0025727300049577.

54 I. Loudon, *Death in Childbirth* (Oxford: Oxford University Press, 1992), https://academic.oup.com/book/8772.

55 Verdoorn, *Het gezondheidswezen*, 206.

56 A. Janssens, O. Lammertink, T. Riswick, S. Muurling, E. Kuiper, M. Twijnstra, Amsterdam Cause-of-death Database, 1854-1940, version 1.0, 2023, Unpublished dataset Radboud Group for Historical Demography and Family History, Radboud University, The Netherlands.

57 M. G. Neurdenburg, *Doodsoorzaak en statistiek* (Leiden University, 1929).

58 Janssens and Riswick, ‘What Was Killing Babies in Amsterdam?’, 238.

59 ‘Bevolking-register der stad Amsterdam, 1851–1853’, 1853, No. 5000, inv.no. 258–994, Amsterdam City Archive, https://archief.amsterdam/inventarissen/details/5000.

60 Ekamper and van Poppel, ‘Infant Mortality’, 5.

61 Tax values are derived from taxable incomes recorded in the cadaster of 1832 as collected by https://hisgis.nl/projecten/amsterdam/. They are considered among the best available data to reconstruct SES on house-level and remained fairly representative until the second half of the nineteenth century. See Owen Lammertink, *De opkomst van het moderne ziektepatroon? Doodsoorzaken, degeneratieve aandoeningen en sociale ongelijkheid in Amsterdam, 1854–1926* (Nijmegen: Radboud University, 2023), 84, https://repository.ubn.ru.nl/handle/2066/292090.

62 M. Raat and R. L. Zijdeman, *HISGIS Amsterdam Location Points* (IISH Data Collection, 2019), https://datasets.iisg.amsterdam/dataset.xhtml?persistentId=hdl:10622/FHJJYK.

63 ‘Algemeen adresboek der stad Amsterdam voor de jaren 1855–1856’, 1856, No. 30274, inv.no. 16, Amsterdam City Archive, https://archief.amsterdam/inventarissen/file/d751dead1c258b435be991c7deacddd9.

64 van Lieburg and Marland, ‘Midwife Regulation, Education, and Practice in The Netherlands during the Nineteenth Century.’

65 Only two deaths (0.1 per cent) from the 1598 cause-of-death records could not be linked to a death certificate, but could be linked to a birth certificate with age at death, sex, and residential address. Twenty-seven infant deaths which could not be linked to a birth certificate were added to the births (which is 1.6 per cent of all deaths and 0.3 per cent of all births).

66 Since there is a time gap of several years between the births and the population register, we linked combinations (in decreasing importance) of family and first names (including spelling variants), approximate ages (corrected for the time gap), occupations and residential addresses. We successfully matched 65.7 per cent of all births to both parents, 11.3 per cent to the father and 3.4 per cent to the mother (0.3 per cent to single mothers).

67 R. Mourits, I. K. van Dijk, and Kees Mandemakers, ‘From Matched Certificates to Related Persons’, *Historical Life Course Studies*, 2020, 9, 52, https://doi.org/10.51964/hlcs9310.

68 J. Kok, *Langs verboden wegen: De achtergronden van buitenechtelijke geboorten in Noord-Holland 1812–1914* (Hilversum: Verloren, 1991), 33.

69 D. Cox, ‘Regression Models and Life Tables (with Discussion)’, *Journal of the Royal Statistical Society, Series B*, 1972, 34, 187–220.

70 Mühlichen, Scholz, and Doblhammer, ‘Social Differences in Infant Mortality’, 195.

71 Ward, ‘Perinatal Mortality’; Mühlichen, ‘Premature Mortality’, 34; Jaadla and Puur, ‘The Impact of Water Supply and Sanitation’, 173.

72 J. P. Fine and R. J. Gray, ‘A Proportional Hazards Model for the Subdistribution of a Competing Risk’, *Journal of the American Statistical Association*, 1999, 94, 496–509, https://doi.org/10.1080/01621459.1999.10474144.

73 Ekamper and van Poppel, ‘Infant Mortality’, 7.

74 B. Van De Putte and A. Miles, ‘A Social Classification Scheme for Historical Occupational Data’, *Historical Methods: A Journal of Quantitative and Interdisciplinary History*, 2005, 38, 61–94, https://doi.org/10.3200/HMTS.38.2.61-94.

75 H. Laloli, ‘Buurt- en wijkindeling Amsterdam in 1850’, Amsterdamhistorie, accessed May 25, 2022, http://amsterdamhistorie.nl/buurten/buurten1850.html.

76 Janssens, ‘Constructing SHiP’, 12–13.

77 For an overview of the disease terms belonging to the different infantcat groupings, see appendix 14 in Janssens and Riswick, ‘What Was Killing Babies in Amsterdam?’, 277–79.

78 R. J. van der Bie and J. P. Smits, eds., *Tweehonderd jaar statistiek in tijdreeksen, 1800–1999* (Voorburg: Centraal Bureau voor de Statistiek, 2001), 14.

79 In the period 1841–1860, infant mortality rates in the West of the Netherlands were on average 30 per cent higher than the national average. In this period, the infant mortality rate of Amsterdam was 20 per cent higher than the national average and 12 per cent higher than in the surrounding rural areas (calculated from P. Ekamper and F. W. A. van Poppel, *Infant Mortality by Municipality in the Netherlands, 1841-1939* (DANS Data Station Social Sciences and Humanities, 2008), https://doi.org/10.17026/dans-xz2-mya6. In 1856, the difference between Amsterdam and the national level was smaller, but still significant.

80 D. R. Farinas and M. Oris, ‘New Approaches to Death in Cities during the Health Transition: An Introduction’, in D. R. Farinas and M. Oris, eds, *New Approaches to Death in Cities during the Health Transition* (New York: Springer, 2016), 5–7; R. Schofield, D. Reher, and A. Bideau, eds., *The Decline of Mortality in Europe: International Studies in Demography* (Oxford: Clarendon Press, 1991); F. Van de Walle, ‘Infant Mortality and the European Demographic Transition’, in A. J. Coale and S. C. Watkins, eds, *The Decline of Fertility in Europe* (Princeton: Princeton University Press, 1986); L. Cain and S. C. Hong, ‘Survival in 19th Century Cities: The Larger the City, the Smaller Your Chances’, *Explorations in Economic History*, October 2009, 46, 450–63, https://doi.org/10.1016/j.eeh.2009.05.001.

81 Ekamper and van Poppel, ‘Infant Mortality’, 14.

82 G. Mooney, ‘Shifting Sex Differentials in Mortality during Urban Epidemiological Transition: The Case of Victorian London’, *International Journal of Population Geography*, 2002, 8, 20, https://doi.org/10.1002/ijpg.233; K. Tymicki, ‘The Correlates of Infant and Childhood Mortality: A Theoretical Overview and New Evidence from the Analysis of Longitudinal Data of the Bejsce (Poland) Parish Register Reconstitution Study of the 18th–20th Centuries’, *Demographic Research*, 2009, 20, 580–86, https://doi.org/10.4054/DemRes.2009.20.23; Jaadla and Puur, ‘The Impact of Water Supply and Sanitation’, 172; R. Derosas, ‘Watch Out for the Children! Differential Infant Mortality of Jews and Catholics in Nineteenth-Century Venice’, *Historical Methods: A Journal of Quantitative and Interdisciplinary History*, 2003, 36, 119–21, https://doi.org/10.1080/01615440309601605.

83 Mühlichen, ‘Premature Mortality’, 35; S. H. Preston and M. R. Haines, *Fatal Years: Child Mortality in Late Nineteenth-Century America* (Princeton: Princeton University Press, 1991), 30; J. P. Vögele, ‘Urban Infant Mortality in Imperial Germany’, *Social History of Medicine*, 1994, 7, 401–25, https://doi.org/10.1093/shm/7.3.401; J. Kok, F. W. A. van Poppel, and E. Kruse, ‘Mortality among Illegitimate Children in Mid-Nineteenth-Century the Hague’, in C. A. Corsini and P. P. Viazzo, eds, *The Decline of Infant and Child Mortality. The European Experience: 1750–1990* (The Hague: The Hague: Martinus Nijhoff Publishers, 1997), 193–94.

84 Ekamper and van Poppel, ‘Infant Mortality’, 11–13.

85 Riswick, Muurling, and Buzasi, ‘Exploring the Mortality Advantage’, 724.

86 For recent overviews of the debate, see Mühlichen, ‘Premature Mortality’, 35; Farinas and Oris, ‘New Approaches to Death in Cities.’

87 T. Bengtsson, ‘The Vulnerable Child: Economic Insecurity and Child Mortality in Pre-Industrial Sweden: A Case Study of Västanfors, 1757–1850’, *European Journal of Population/Revue Européenne de Démographie*, 1999, 15, 117–51; Breschi et al., ‘Socioeconomic Conditions, Health and Mortality’; R. I. Woods, P. A. Watterson, and J. H. Woodward, ‘The Causes of Rapid Infant Mortality Decline in England and Wales, 1861–1921 Part I’, *Population Studies*, 1988, 42, 343–66; Derosas, ‘Watch Out for the Children!’; Molitoris, ‘Disparities in Death’; Mühlichen, Scholz, and Doblhammer, ‘Social Differences in Infant Mortality’; L. Kesztenbaum and J. L. Rosenthal, ‘The Democratization of Longevity: How the Poor Became Old in Paris, 1880–1913’, in D. Ramiro Fariñas and M. Oris, eds, *New Approaches to Death in Cities during the Health Transition* (Cham: Springer International Publishing, 2016), 137–54, https://doi.org/10.1007/978-3-319-43002-7_8; F. W. A. van Poppel, M. Jonker, and K. Mandemakers, ‘Differential Infant and Child Mortality in Three Dutch Regions, 1812–1909’, *Economic History Review*, 2005, 58, 272–309, https://doi.org/10.1111/j.1468-0289.2005.00305.x.

88 S. Scott and C. J. Duncan, ‘Interacting Effects of Nutrition and Social Class Differentials on Fertility and Infant Mortality in a Pre-Industrial Population’, *Population Studies*, 2000, 54, 71–87, https://doi.org/10.1080/713779065; Mühlichen, ‘Premature Mortality’, 35; Molitoris, ‘Disparities in Death’, 459.

89 van Lieburg and Marland, ‘Midwife Regulation, Education, and Practice in The Netherlands during the Nineteenth Century.’

90 Jaadla and Puur, ‘The Impact of Water Supply and Sanitation’; van Poppel and van der Heijden, ‘The Effects of Water Supply on Infant and Childhood Mortality’; Kesztenbaum and Rosenthal, ‘The Democratization of Longevity’; Molitoris, ‘Disparities in Death’, 459.

91 Egeling, ‘Bijdrage tot de kennis’, 588.

92 Mühlichen, ‘Premature Mortality’, 65, 71; Derosas, ‘Watch Out for the Children!’, 122.

93 Mühlichen, ‘Premature Mortality’, 35; Woods, Watterson, and Woodward, ‘The Causes of Rapid Infant Mortality Decline I.’

94 Molitoris, ‘Disparities in Death’, 468.

95 Janssens and Riswick, ‘What Was Killing Babies in Amsterdam?’; N. J. M. van den Boomen, ‘Born Close to Death. Region, Roman Catholicism and Infant Mortality in the Netherlands, 1875–1899’ (Dissertation, Radboud University, 2021), 14, https://repository.ubn.ru.nl/handle/2066/240453.

96 See Supplementary Figure S3.

97 Mühlichen, ‘Premature Mortality’, 34.

98 For a recent overview of the debate and contemporary medical knowledge, see M. Murkens, B. Pelzer, and A. Janssens, ‘Transitory Inequalities: How Individual-Level Cause-Specific Death Data Can Unravel Socioeconomic Inequalities in Infant Mortality in Maastricht, the Netherlands, 1864–1955’, *The History of the Family*, 2022, 11, https://doi.org/10.1080/1081602X.2022.2084442.

99 Molitoris, ‘Disparities in Death’, 468; van den Boomen, ‘Born Close to Death’, 40; Murkens, Pelzer, and Janssens, ‘Transitory Inequalities’, 11; Jaadla and Puur, ‘The Impact of Water Supply and Sanitation’, 174.

100 Janssens and Riswick, ‘What Was Killing Babies in Amsterdam?’

101 F. W. A. van Poppel and J. P. van Dijk, ‘The Development of Cause-of-Death Registration in the Netherlands, 1865–1955’, *Continuity and Change*, 1997, 12, 265–87, https://doi.org/10.1017/S0268416097002932; van den Boomen, ‘Born Close to Death.’

102 For the full multivariate Fine‐Grey competing risk subdistribution hazard model estimations, see the supplementary Tables S1 to S5.

103 Ward, ‘Perinatal Mortality’; R. Millward and F. Bell, ‘Infant Mortality in Victorian Britain: The Mother as Medium’, *The Economic History Review*, 2001, 54, 699–733, https://doi.org/10.1111/1468-0289.00209; R. Floud et al., *The Changing Body: Health, Nutrition, and Human Development in the Western World since 1700* (Cambridge University Press, 2011), 12–16, https://doi.org/10.1017/CBO9780511975912.

104 Janssens and Riswick, ‘What Was Killing Babies in Amsterdam?’

105 A similar argument is made for the high portions of ‘unknowns’ in Scotland by A. Reid and E. Garrett, ‘Doctors and the Causes of Neonatal Death in Scotland in the Second Half of the Nineteenth Century’, *Annales de Démographie Historique*, 2012, 123, 173, https://doi.org/10.3917/adh.123.0149.

106 Mackenbach, *A History of Population Health*, 189.

107 S. J. Goerg and J. Kaiser, ‘Nonparametric Testing of Distributions—the Epps–Singleton Two-Sample Test Using the Empirical Characteristic Function’, *The Stata Journal*, 2009, 9, 454–65, https://doi.org/10.1177/1536867X0900900307.

108 C. Lesger and M. H. D. Van Leeuwen, ‘Residential Segregation from the Sixteenth to the Nineteenth Century: Evidence from the Netherlands’, *The Journal of Interdisciplinary History*, 2012, 42, 366, https://doi.org/10.1162/JINH_a_00254.

109 The mortality advantage of Jewish neighbourhoods and its potential causes are explored in more detail in Riswick, Muurling, and Buzasi, ‘Exploring the Mortality Advantage.’

110 Bengtsson, ‘The Vulnerable Chilld’, 126; Millward and Bell, ‘The Mother as Medium’, 705.

111 Janssens and Riswick, ‘What Was Killing Babies in Amsterdam?’; Floud et al., *The Changing Body*, 12–16.

112 E. C. Walhout, ‘Is Breast Best? Evaluating Breastfeeding Patterns and Causes of Infant Death in a Dutch Province in the Period 1875–1900’, *The History of the Family*, 15 March 2010, 15, 76–90, https://doi.org/10.1016/j.hisfam.2009.12.001.

113 Millward and Bell, ‘The Mother as Medium’, 716–17, 27.

114 Millward and Bell, 716–18, 27; C. Holdsworth, ‘Women’s Work and Family Health: Evidence from the Staffordshire Potteries, 1890–1920’, *Continuity and Change*, 1997, 12, 103–28, https://doi.org/10.1017/S026841609700283X; E. Garrett and A. Reid, ‘Satanic Mills, Pleasant Lands: Spatial Variation in Women’s Work, Fertility and Infant Mortality as Viewed from the 1911 Census’, *Historical Research*, 1994, 67, 156–77, https://doi.org/10.1111/j.1468-2281.1994.tb01822.x; Eilidh Garrett and Alice Reid, ‘Thinking of England and Taking Care: Family Building Strategies and Infant Mortality in England and Wales, 1891–1911’, *International Journal of Population Geography*, September 1995, 1, 69–102, https://doi.org/10.1002/ijpg.6060010106.

115 M. H. D. van Leeuwen, ‘Surviving with a Little Help: The Importance of Charity to the Poor of Amsterdam 1800–50, in a Comparative Perspective’, *Social History*, 1993, 18, 235; A. Knotter, ‘Stedelijke economie en arbeidsmarkt: Amsterdam in de eerste helft van de negentiende eeuw’, *BMGN - Low Countries Historical Review*, 1986, 101, 575.

116 Ekamper and van Poppel, ‘Infant Mortality’, 9–10, 14.

117 A. H. Israëls, ‘De sterfte der kinderen in de drie eerste jaren des levens te Amsterdam, in de jaren 1850–1859’, *Nederlands tijdschrift voor geneeskunde*, 1862, 6, 299.

118 Reid and Garrett, ‘Doctors and the Causes of Neonatal Death’, 174.

119 V. A. Luque de Haro, J. M. Pujadas-Mora, and J. J. García-Gómez, ‘Inequality in Mortality in Pre-Industrial Southern Europe during an Epidemic Episode: Socio-Economic Determinants (Eighteenth - Nineteenth Centuries Spain)’, *Economics & Human Biology*, 2021, 40, 13, https://doi.org/10.1016/j.ehb.2020.100941.

120 Bengtsson and van Poppel, ‘Socioeconomic Inequalities in Death.’

121 A. R. Omran, ‘The Epidemiologic Transition: A Theory of the Epidemiology of Population Change’, *The Milbank Quarterly*, December 2005, 83, 737–38, 744–47, https://doi.org/10.1111/j.1468-0009.2005.00398.x; van Poppel, ‘Het komen en gaan van ziekten’, 6.

122 Ekamper and van Poppel, ‘Infant Mortality’, 14; Mooney, ‘Shifting Sex Differentials in Mortality’, 20; Tymicki, ‘The Correlates of Infant and Childhood Mortality’, 580–86; Jaadla et al., ‘Infant and Child Mortality by Socio-Economic Status’, 172; Derosas, ‘Watch Out for the Children!’, 119–21; Mühlichen, ‘Premature Mortality’, 35; Kok, van Poppel, and Kruse, ‘Mortality among Illegitimate Children’, 193–94; Walhout, ‘An Infants’ Graveyard?’, 83–90; Riswick, Muurling, and Buzasi, ‘Exploring the Mortality Advantage.’

123 Ward, ‘Perinatal Mortality’, 394.

124 Ward, ‘Perinatal Mortality’; Millward and Bell, ‘The Mother as Medium’; Floud et al., *The Changing Body*, 12–16; Janssens and Riswick, ‘What Was Killing Babies in Amsterdam?’

## Notes

Sanne Muurling is Assistant Professor in social history at Radboud University. Her work focuses on the history of everyday life, gender and socioeconomic inequalities between 1600 and 1900, particularly in urban spaces. She has published on topics such as crime and deviance, and social and economic inequalities in death. Her monograph, *Everyday crime, Criminal Justice and Gender in Early Modern Bologna* was published in 2021.

Peter Ekamper is Senior Researcher at the Netherlands Interdisciplinary Demographic Institute (NIDI-KNAW). He holds a PhD in Spatial Sciences from the University of Groningen on time and space in historical demography and specialises in the field of historical demography, spatial demography and population projections. He is an editor of the NIDI popular science journal Demos, Bulletin on Population and Society.

