# Supplementary material for: A World of (In)difference? Social Inequalities Among Infants’ Causes of Death in Mid-nineteenth-Century Amsterdam
Source: Soc Hist Med. 2024 Nov 21;38(2):291–322. doi: 10.1093/shm/hkae066 (PMC12264207; doi:10.1093/shm/hkae066)
Supplement: hkae066_suppl_Supplementary_Material [file hkae066_suppl_supplementary_material.docx]

**Supplementary figures and tables**

Belonging to Sanne Muurling and Peter Ekamper, ‘A World of (In)difference? Social Inequalities Among Infants’ Causes of Death in Mid-nineteenth-Century Amsterdam’

Figure S1. Subdivision of Amsterdam’s administrative neighbourhoods into eight largely socio-economically homogenous districts, c. 1856


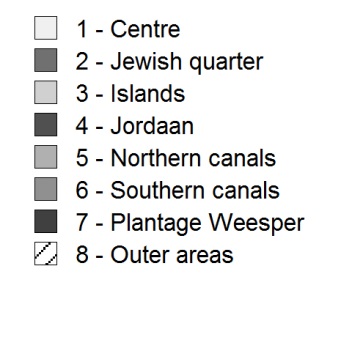

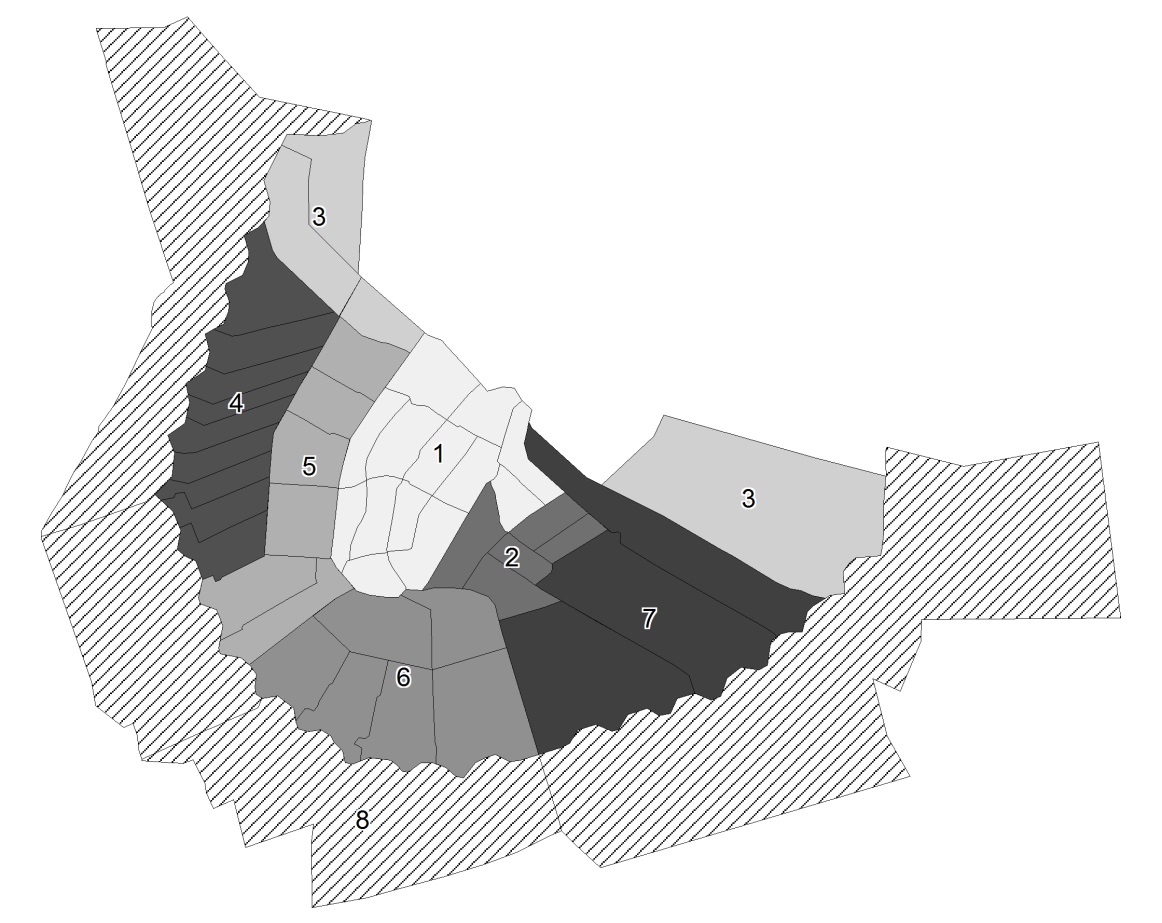


Source: Condensed version of the categorization of neighbourhoods by H. Laloli, “Buurt- en wijkindeling Amsterdam in 1850,” AmsterdamHistorie, accessed May 25, 2022, http://amsterdamhistorie.nl/buurten/buurten1850.html

Figure S2. Infant deaths by age at death, Amsterdam, children born in 1856


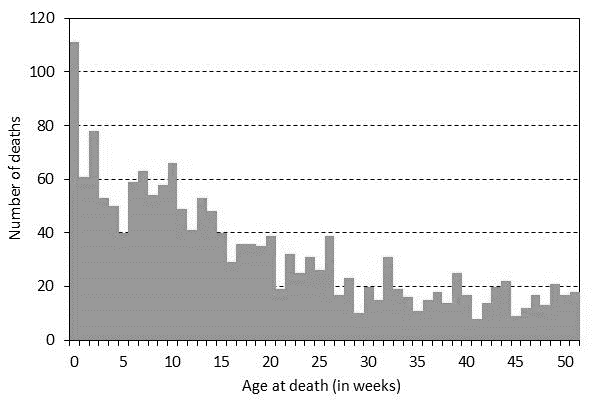

Sources: Own calculations based on linked data from Amsterdam Cause-of-death Database, Amsterdam birth (1856) and death (1856-57) certificates.

Figure S3. Fine-Gray competing risk model subdistribution hazard ratios (SHR) for infant mortality by age at death and cause of death, condensed results for social class only, Amsterdam, children born in 1856

Notes: Be aware of the different y-scale for figure (e); neonatal = first 28 days, early post neonatal = from day 29 up to the 6^th^ month, later post neonatal = from the 7^th^ up to the 12^th^ month; (ref.) = reference category; for the full multivariate Fine‐Gray competing risk subdistribution hazard model estimations see Annex tables …; Cox all-causes model estimates (table 1, models 2-5) added for comparison reasons.
Sources: Own calculations based on linked data from Amsterdam Cause-of-death Database, Amsterdam birth (1856) and death (1856-57) certificates, Amsterdam population register 1851-1853, and HISGIS Amsterdam.

Table S1. Fine-Gray competing risk model subdistribution hazard ratios (SHR) for infant mortality from infectious diseases in Amsterdam, children born in 1856

|  |  | Mulltivariate models | | | | | | | | | | | |
| --- | --- | --- | --- | --- | --- | --- | --- | --- | --- | --- | --- | --- | --- |
|  |  | Total | | | Neonatal | | | Early post neonatal | | | Later post neonatal | | |
|  |  | SHR | P>\|z\| |  | SHR | P>\|z\| |  | SHR | P>\|z\| |  | SHR | P>\|z\| |  |
|  |  |  |  |  |  |  |  |  |  |  |  |  |  |
| Sex | Males | 1.234 | 0.032 | ** | 1.740 | 0.212 |  | 1.307 | 0.070 | * | 1.072 | 0.650 |  |
|  |  |  |  |  |  |  |  |  |  |  |  |  |  |
| Age mother | |  |  |  |  |  |  |  |  |  |  |  |  |
|  | 15-19 | 1.438 | 0.447 |  | - |  |  | 3.104 | 0.059 | * | - |  |  |
|  | 20-24 | (ref.) |  |  | (ref.) |  |  | (ref.) |  |  | (ref.) |  |  |
|  | 25-29 | 0.942 | 0.821 |  | 0.458 | 0.261 |  | 0.912 | 0.823 |  | 1.148 | 0.749 |  |
|  | 30-34 | 1.391 | 0.182 |  | 0.342 | 0.159 |  | 1.064 | 0.873 |  | 2.040 | 0.082 | * |
|  | 35-39 | 1.258 | 0.376 |  | 0.306 | 0.131 |  | 1.226 | 0.615 |  | 1.714 | 0.204 |  |
|  | 40+ | 1.713 | 0.051 | * | 0.427 | 0.414 |  | 1.665 | 0.229 |  | 2.104 | 0.107 |  |
|  | Unknown | 0.918 | 0.879 |  | 0.043 | 0.073 | * | 1.133 | 0.894 |  | 1.494 | 0.636 |  |
|  |  |  |  |  |  |  |  |  |  |  |  |  |  |
| Age difference father | |  |  |  |  |  |  |  |  |  |  |  |  |
|  | Younger (2+ years) | 0.896 | 0.493 |  | 0.856 | 0.832 |  | 1.109 | 0.670 |  | 0.642 | 0.093 | * |
|  | Equal (-2 to +2 years) | 0.950 | 0.714 |  | 0.682 | 0.559 |  | 1.068 | 0.764 |  | 0.990 | 0.960 |  |
|  | Older (2-9 years) | (ref.) |  |  | (ref.) |  |  | (ref.) |  |  | (ref.) |  |  |
|  | Much older (9+ years) | 0.963 | 0.843 |  | 1.519 | 0.533 |  | 0.853 | 0.620 |  | 1.057 | 0.847 |  |
|  | Unknown | 0.728 | 0.416 |  | 0.388 | 0.652 |  | 0.583 | 0.405 |  | 0.781 | 0.682 |  |
|  |  |  |  |  |  |  |  |  |  |  |  |  |  |
| Multiple births | | 1.172 | 0.553 |  | - |  |  | 2.614 | 0.002 | *** | 1.181 | 0.749 |  |
|  |  |  |  |  |  |  |  |  |  |  |  |  |  |
| Religion | |  |  |  |  |  |  |  |  |  |  |  |  |
|  | Dutch Reformed | (ref.) |  |  | (ref.) |  |  | (ref.) |  |  | (ref.) |  |  |
|  | Other Protestant | 1.073 | 0.638 |  | 1.072 | 0.916 |  | 0.701 | 0.172 |  | 1.444 | 0.086 | * |
|  | Catholic | 1.007 | 0.964 |  | 1.866 | 0.268 |  | 0.997 | 0.990 |  | 0.876 | 0.573 |  |
|  | Jewish | 1.165 | 0.524 |  | 0.917 | 0.918 |  | 0.825 | 0.663 |  | 1.291 | 0.476 |  |
|  | Unknown | 1.895 | 0.096 | * | 7.782 | 0.014 | ** | 1.887 | 0.283 |  | 1.369 | 0.561 |  |
|  |  |  |  |  |  |  |  |  |  |  |  |  |  |
| Social class | |  |  |  |  |  |  |  |  |  |  |  |  |
|  | Elite | 1.121 | 0.735 |  | - |  |  | 0.527 | 0.388 |  | 1.763 | 0.159 |  |
|  | Middle class | 0.778 | 0.097 | * | 1.406 | 0.624 |  | 0.799 | 0.349 |  | 0.623 | 0.043 | ** |
|  | Skilled | 0.782 | 0.056 | * | 0.647 | 0.455 |  | 0.886 | 0.541 |  | 0.622 | 0.018 | ** |
|  | Semi-skilled | 0.618 | 0.011 | ** | - |  |  | 0.565 | 0.059 | * | 0.657 | 0.134 |  |
|  | Unskilled | (ref.) |  |  | (ref.) |  |  | (ref.) |  |  | (ref.) |  |  |
|  | Unknown | 0.554 | 0.078 | * | 1.246 | 0.667 |  | 0.703 | 0.505 |  | 0.325 | 0.047 | ** |
|  |  |  |  |  |  |  |  |  |  |  |  |  |  |
| Single mother | | 2.639 | 0.006 | *** | 5.689 | 0.210 |  | 2.466 | 0.087 | * | 4.269 | 0.017 | ** |
|  |  |  |  |  |  |  |  |  |  |  |  |  |  |
| Season of birth | |  |  |  |  |  |  |  |  |  |  |  |  |
|  | Spring | (ref.) |  |  | (ref.) |  |  | (ref.) |  |  | (ref.) |  |  |
|  | Summer | 0.838 | 0.198 |  | 1.631 | 0.381 |  | 0.676 | 0.061 | * | 1.213 | 0.393 |  |
|  | Autumn | 0.886 | 0.360 |  | 1.162 | 0.809 |  | 0.527 | 0.003 | *** | 1.504 | 0.053 | * |
|  | Winter | 0.830 | 0.168 |  | 0.758 | 0.689 |  | 0.828 | 0.329 |  | 1.055 | 0.814 |  |
|  |  |  |  |  |  |  |  |  |  |  |  |  |  |
| Tax value | |  |  |  |  |  |  |  |  |  |  |  |  |
|  | <100 | (ref.) |  |  | (ref.) |  |  | (ref.) |  |  | (ref.) |  |  |
|  | 100-200 | 1.266 | 0.067 | * | 0.735 | 0.587 |  | 1.451 | 0.064 | * | 1.284 | 0.210 |  |
|  | 200+ | 1.115 | 0.503 |  | 0.640 | 0.532 |  | 1.492 | 0.112 |  | 0.969 | 0.899 |  |
|  |  |  |  |  |  |  |  |  |  |  |  |  |  |
|  |  |  |  |  |  |  |  |  |  |  |  |  |  |
| Backstreet alley | | 1.087 | 0.579 |  | 0.245 | 0.186 |  | 1.537 | 0.041 | ** | 0.871 | 0.597 |  |
|  |  |  |  |  |  |  |  |  |  |  |  |  |  |
| Distance to midwife | |  |  |  |  |  |  |  |  |  |  |  |  |
|  | <75 | (ref.) |  |  | (ref.) |  |  | (ref.) |  |  | (ref.) |  |  |
|  | 75-150 | 0.846 | 0.191 |  | 0.520 | 0.264 |  | 0.635 | 0.024 | ** | 0.951 | 0.799 |  |
|  | 150-300 | 0.980 | 0.871 |  | 0.938 | 0.908 |  | 1.060 | 0.757 |  | 1.013 | 0.950 |  |
|  | 300-600 | 0.852 | 0.530 |  | 0.882 | 0.905 |  | 1.096 | 0.804 |  | 0.585 | 0.202 |  |
|  | 600+ | 0.757 | 0.706 |  | - |  |  | 2.413 | 0.325 |  | - |  |  |
|  |  |  |  |  |  |  |  |  |  |  |  |  |  |
|  |  |  |  |  |  |  |  |  |  |  |  |  |  |
| Water quality canals | |  |  |  |  |  |  |  |  |  |  |  |  |
|  | Seafront | (ref.) |  |  | (ref.) |  |  | (ref.) |  |  | (ref.) |  |  |
|  | "Best" | 0.734 | 0.379 |  | - |  |  | 0.409 | 0.109 |  | 1.259 | 0.638 |  |
|  | "Intermediate" | 0.736 | 0.045 | ** | 0.475 | 0.249 |  | 0.723 | 0.160 |  | 0.682 | 0.108 |  |
|  | "Worst" | 0.892 | 0.620 |  | 0.716 | 0.766 |  | 0.690 | 0.267 |  | 1.082 | 0.821 |  |
|  |  |  |  |  |  |  |  |  |  |  |  |  |  |
|  |  |  |  |  |  |  |  |  |  |  |  |  |  |
| House in front of canal | | 0.880 | 0.314 |  | 0.793 | 0.682 |  | 0.680 | 0.061 | * | 1.223 | 0.279 |  |
|  |  |  |  |  |  |  |  |  |  |  |  |  |  |
| Neighborhood | |  |  |  |  |  |  |  |  |  |  |  |  |
|  | Centre | (ref.) |  |  | (ref.) |  |  | (ref.) |  |  | (ref.) |  |  |
|  | Jewish quarter | 0.640 | 0.062 | * | 0.309 | 0.147 |  | 0.385 | 0.029 | ** | 0.666 | 0.272 |  |
|  | Islands | 0.715 | 0.118 |  | 1.221 | 0.792 |  | 0.372 | 0.009 | *** | 0.940 | 0.838 |  |
|  | Jordaan | 0.823 | 0.430 |  | 0.558 | 0.605 |  | 0.937 | 0.856 |  | 0.655 | 0.267 |  |
|  | Northern canals | 0.988 | 0.958 |  | 0.518 | 0.644 |  | 1.193 | 0.568 |  | 0.785 | 0.500 |  |
|  | Southern canals | 0.976 | 0.916 |  | 0.964 | 0.974 |  | 1.133 | 0.691 |  | 0.741 | 0.410 |  |
|  | Plantage Weesper | 0.785 | 0.255 |  | 0.322 | 0.298 |  | 0.827 | 0.525 |  | 0.607 | 0.159 |  |
|  | Outer areas | 1.489 | 0.375 |  | - |  |  | 2.104 | 0.275 |  | 1.681 | 0.392 |  |
|  |  |  |  |  |  |  |  |  |  |  |  |  |  |

Notes: Neonatal = first 28 days, early post neonatal = from day 29 up to the 6^th^ month, later post neonatal = from the 7^th^ up to the 12^th^ month; significance * p<0.1, ** p<0.01, *** p<0.001; (ref.) = reference category; - = omitted variable categories.
Sources: Own calculations based on linked data from Amsterdam Cause-of-death Database, Amsterdam birth (1856) and death (1856-57) certificates, Amsterdam population register 1851-1853, and HISGIS Amsterdam.

Table S2. Fine-Gray competing risk model subdistribution hazard ratios (SHR) for infant mortality from congenital and birth disorders in Amsterdam, children born in 1856

|  |  | Mulltivariate models | | | | | | | | | | | |
| --- | --- | --- | --- | --- | --- | --- | --- | --- | --- | --- | --- | --- | --- |
|  |  | Total | | | Neonatal | | | Early post neonatal | | | Later post neonatal | | |
|  |  | SHR | P>\|z\| |  | SHR | P>\|z\| |  | SHR | P>\|z\| |  | SHR | P>\|z\| |  |
|  |  |  |  |  |  |  |  |  |  |  |  |  |  |
| Sex | Males | 1.017 | 0.915 |  | 1.003 | 0.992 |  | 1.266 | 0.271 |  | 0.404 | 0.070 | * |
|  |  |  |  |  |  |  |  |  |  |  |  |  |  |
| Age mother | |  |  |  |  |  |  |  |  |  |  |  |  |
|  | 15-19 | 3.521 | 0.225 |  | - |  |  | 3.746 | 0.377 |  | 2.434 | 0.556 |  |
|  | 20-24 | (ref.) |  |  | (ref.) |  |  | (ref.) |  |  | (ref.) |  |  |
|  | 25-29 | 3.843 | 0.064 | * | - |  |  | 4.658 | 0.132 |  | 0.858 | 0.888 |  |
|  | 30-34 | 2.804 | 0.155 |  | - |  |  | 2.468 | 0.379 |  | 0.836 | 0.859 |  |
|  | 35-39 | 3.972 | 0.058 | * | - |  |  | 3.348 | 0.246 |  | 2.354 | 0.380 |  |
|  | 40+ | 6.029 | 0.016 | ** | - |  |  | 5.091 | 0.130 |  | 0.989 | 0.994 |  |
|  | Unknown | 2.191 | 0.417 |  | - |  |  | 6.595 | 0.112 |  | 0.030 | 0.074 | * |
|  |  |  |  |  |  |  |  |  |  |  |  |  |  |
| Age difference father | |  |  |  |  |  |  |  |  |  |  |  |  |
|  | Younger (2+ years) | 1.005 | 0.987 |  | 0.560 | 0.381 |  | 1.175 | 0.697 |  | 0.789 | 0.770 |  |
|  | Equal (-2 to +2 years) | 1.162 | 0.557 |  | 1.668 | 0.248 |  | 1.012 | 0.973 |  | 0.687 | 0.633 |  |
|  | Older (2-9 years) | (ref.) |  |  | (ref.) |  |  | (ref.) |  |  | (ref.) |  |  |
|  | Much older (9+ years) | 1.070 | 0.852 |  | 0.914 | 0.863 |  | 0.691 | 0.519 |  | 2.282 | 0.291 |  |
|  | Unknown | 1.934 | 0.140 |  | 1.723 | 0.401 |  | 0.926 | 0.901 |  | 2.267 | 0.528 |  |
|  |  |  |  |  |  |  |  |  |  |  |  |  |  |
| Multiple births | | 5.681 | 0.000 | *** | 14.754 | 0.000 | *** | 3.851 | 0.001 | *** | 11.930 | 0.000 | *** |
|  |  |  |  |  |  |  |  |  |  |  |  |  |  |
| Religion | |  |  |  |  |  |  |  |  |  |  |  |  |
|  | Dutch Reformed | (ref.) |  |  | (ref.) |  |  | (ref.) |  |  | (ref.) |  |  |
|  | Other Protestant | 1.836 | 0.012 | ** | 3.051 | 0.012 | ** | 1.580 | 0.179 |  | 1.477 | 0.516 |  |
|  | Catholic | 1.843 | 0.009 | *** | 2.925 | 0.013 | ** | 1.574 | 0.165 |  | 1.212 | 0.766 |  |
|  | Jewish | 0.925 | 0.894 |  | 1.048 | 0.960 |  | 0.911 | 0.911 |  | - |  |  |
|  | Unknown | 1.257 | 0.677 |  | 0.621 | 0.520 |  | 0.893 | 0.865 |  | 19.386 | 0.000 | *** |
|  |  |  |  |  |  |  |  |  |  |  |  |  |  |
| Social class | |  |  |  |  |  |  |  |  |  |  |  |  |
|  | Elite | 0.308 | 0.266 |  | 0.674 | 0.765 |  | - |  |  | - |  |  |
|  | Middle class | 0.390 | 0.003 | *** | 0.382 | 0.052 | * | 0.436 | 0.050 | * | 0.248 | 0.247 |  |
|  | Skilled | 0.654 | 0.048 | ** | 0.320 | 0.014 | ** | 0.687 | 0.187 |  | 1.347 | 0.612 |  |
|  | Semi-skilled | 0.594 | 0.082 | * | 0.198 | 0.036 | ** | 0.810 | 0.571 |  | 1.386 | 0.678 |  |
|  | Unskilled | (ref.) |  |  | (ref.) |  |  | (ref.) |  |  | (ref.) |  |  |
|  | Unknown | 0.919 | 0.835 |  | 0.996 | 0.996 |  | 0.618 | 0.428 |  | 2.637 | 0.267 |  |
|  |  |  |  |  |  |  |  |  |  |  |  |  |  |
| Single mother | | 2.170 | 0.073 | * | 1.988 | 0.307 |  | 4.614 | 0.011 | ** | 0.833 | 0.846 |  |
|  |  |  |  |  |  |  |  |  |  |  |  |  |  |
| Season of birth | |  |  |  |  |  |  |  |  |  |  |  |  |
|  | Spring | (ref.) |  |  | (ref.) |  |  | (ref.) |  |  | (ref.) |  |  |
|  | Summer | 1.109 | 0.640 |  | 2.804 | 0.022 | ** | 0.776 | 0.404 |  | 0.661 | 0.509 |  |
|  | Autumn | 0.792 | 0.323 |  | 1.240 | 0.664 |  | 0.604 | 0.111 |  | 0.449 | 0.270 |  |
|  | Winter | 0.913 | 0.684 |  | 0.949 | 0.923 |  | 0.939 | 0.819 |  | 0.971 | 0.957 |  |
|  |  |  |  |  |  |  |  |  |  |  |  |  |  |
| Tax value | |  |  |  |  |  |  |  |  |  |  |  |  |
|  | <100 | (ref.) |  |  | (ref.) |  |  | (ref.) |  |  | (ref.) |  |  |
|  | 100-200 | 1.036 | 0.866 |  | 0.587 | 0.206 |  | 1.137 | 0.633 |  | 1.389 | 0.551 |  |
|  | 200+ | 0.971 | 0.909 |  | 0.805 | 0.607 |  | 0.777 | 0.453 |  | 2.275 | 0.274 |  |
|  |  |  |  |  |  |  |  |  |  |  |  |  |  |
|  |  |  |  |  |  |  |  |  |  |  |  |  |  |
| Backstreet alley | | 1.704 | 0.014 | ** | 0.877 | 0.774 |  | 2.307 | 0.003 | *** | 2.587 | 0.088 | * |
|  |  |  |  |  |  |  |  |  |  |  |  |  |  |
| Distance to midwife | |  |  |  |  |  |  |  |  |  |  |  |  |
|  | <75 | (ref.) |  |  | (ref.) |  |  | (ref.) |  |  | (ref.) |  |  |
|  | 75-150 | 0.925 | 0.719 |  | 0.537 | 0.138 |  | 0.814 | 0.480 |  | 4.301 | 0.052 | * |
|  | 150-300 | 0.950 | 0.819 |  | 0.629 | 0.269 |  | 1.041 | 0.890 |  | 1.366 | 0.709 |  |
|  | 300-600 | 1.508 | 0.253 |  | 0.843 | 0.820 |  | 1.550 | 0.350 |  | 4.655 | 0.091 | * |
|  | 600+ | 2.333 | 0.244 |  | 1.946 | 0.591 |  | 2.333 | 0.340 |  | - |  |  |
|  |  |  |  |  |  |  |  |  |  |  |  |  |  |
|  |  |  |  |  |  |  |  |  |  |  |  |  |  |
| Water quality canals | |  |  |  |  |  |  |  |  |  |  |  |  |
|  | Seafront | (ref.) |  |  | (ref.) |  |  | (ref.) |  |  | (ref.) |  |  |
|  | "Best" | 2.021 | 0.231 |  | 0.344 | 0.488 |  | 2.300 | 0.314 |  | 6.707 | 0.094 | * |
|  | "Intermediate" | 1.563 | 0.157 |  | 1.017 | 0.975 |  | 2.172 | 0.071 | * | 0.973 | 0.977 |  |
|  | "Worst" | 1.764 | 0.156 |  | 1.008 | 0.992 |  | 2.492 | 0.089 | * | 2.672 | 0.313 |  |
|  |  |  |  |  |  |  |  |  |  |  |  |  |  |
|  |  |  |  |  |  |  |  |  |  |  |  |  |  |
| House in front of canal | | 1.189 | 0.386 |  | 0.955 | 0.902 |  | 1.649 | 0.062 | * | 0.325 | 0.102 |  |
|  |  |  |  |  |  |  |  |  |  |  |  |  |  |
| Neighborhood | |  |  |  |  |  |  |  |  |  |  |  |  |
|  | Centre | (ref.) |  |  | (ref.) |  |  | (ref.) |  |  | (ref.) |  |  |
|  | Jewish quarter | 0.458 | 0.093 | * | 1.572 | 0.483 |  | 0.163 | 0.013 | ** | - |  |  |
|  | Islands | 0.679 | 0.366 |  | 0.494 | 0.425 |  | 0.963 | 0.944 |  | 0.299 | 0.306 |  |
|  | Jordaan | 1.254 | 0.561 |  | 1.597 | 0.526 |  | 1.068 | 0.900 |  | 0.689 | 0.669 |  |
|  | Northern canals | 1.041 | 0.919 |  | 2.180 | 0.291 |  | 0.852 | 0.743 |  | - |  |  |
|  | Southern canals | 0.736 | 0.396 |  | 1.192 | 0.814 |  | 0.457 | 0.144 |  | 0.621 | 0.501 |  |
|  | Plantage Weesper | 0.390 | 0.094 | * | - |  |  | 0.647 | 0.465 |  | - |  |  |
|  | Outer areas | 1.322 | 0.623 |  | 3.420 | 0.283 |  | 1.103 | 0.894 |  | 1.610 | 0.675 |  |
|  |  |  |  |  |  |  |  |  |  |  |  |  |  |

Notes: Neonatal = first 28 days, early post neonatal = from day 29 up to the 6^th^ month, later post neonatal = from the 7^th^ up to the 12^th^ month; significance * p<0.1, ** p<0.01, *** p<0.001; (ref.) = reference category; - = omitted variable categories.
Sources: Own calculations based on linked data from Amsterdam Cause-of-death Database, Amsterdam birth (1856) and death (1856-57) certificates, Amsterdam population register 1851-1853, and HISGIS Amsterdam.

Table S3. Fine-Gray competing risk model subdistribution hazard ratios (SHR) for infant mortality from weakness in Amsterdam, children born in 1856

|  |  | Mulltivariate models | | | | | | | | | | | |
| --- | --- | --- | --- | --- | --- | --- | --- | --- | --- | --- | --- | --- | --- |
|  |  | Total | | | Neonatal | | | Early post neonatal | | | Later post neonatal | | |
|  |  | SHR | P>\|z\| |  | SHR | P>\|z\| |  | SHR | P>\|z\| |  | SHR | P>\|z\| |  |
|  |  |  |  |  |  |  |  |  |  |  |  |  |  |
| Sex | Males | 1.047 | 0.679 |  | 1.080 | 0.764 |  | 1.109 | 0.475 |  | 0.832 | 0.474 |  |
|  |  |  |  |  |  |  |  |  |  |  |  |  |  |
| Age mother | |  |  |  |  |  |  |  |  |  |  |  |  |
|  | 15-19 | 1.027 | 0.967 |  | - |  |  | 1.217 | 0.805 |  | 4.000 | 0.330 |  |
|  | 20-24 | (ref.) |  |  | (ref.) |  |  | (ref.) |  |  | (ref.) |  |  |
|  | 25-29 | 1.326 | 0.378 |  | 1.072 | 0.915 |  | 0.997 | 0.994 |  | 4.845 | 0.130 |  |
|  | 30-34 | 1.353 | 0.339 |  | 0.744 | 0.667 |  | 1.288 | 0.510 |  | 3.747 | 0.209 |  |
|  | 35-39 | 1.201 | 0.578 |  | 0.568 | 0.418 |  | 1.280 | 0.537 |  | 2.511 | 0.394 |  |
|  | 40+ | 1.497 | 0.259 |  | 0.803 | 0.766 |  | 1.172 | 0.732 |  | 6.258 | 0.092 | * |
|  | Unknown | 4.175 | 0.046 | ** | - |  |  | 2.246 | 0.314 |  | - |  |  |
|  |  |  |  |  |  |  |  |  |  |  |  |  |  |
| Age difference father | |  |  |  |  |  |  |  |  |  |  |  |  |
|  | Younger (2+ years) | 1.149 | 0.461 |  | 1.664 | 0.248 |  | 1.066 | 0.792 |  | 1.315 | 0.522 |  |
|  | Equal (-2 to +2 years) | 0.868 | 0.410 |  | 1.383 | 0.452 |  | 0.777 | 0.248 |  | 0.946 | 0.886 |  |
|  | Older (2-9 years) | (ref.) |  |  | (ref.) |  |  | (ref.) |  |  | (ref.) |  |  |
|  | Much older (9+ years) | 0.990 | 0.965 |  | 1.762 | 0.248 |  | 0.689 | 0.258 |  | 1.770 | 0.216 |  |
|  | Unknown | 0.273 | 0.022 | ** | - |  |  | 0.454 | 0.182 |  | - |  |  |
|  |  |  |  |  |  |  |  |  |  |  |  |  |  |
| Multiple births | | 4.065 | 0.000 | *** | 11.188 | 0.000 | *** | 3.756 | 0.000 | *** | 1.963 | 0.371 |  |
|  |  |  |  |  |  |  |  |  |  |  |  |  |  |
| Religion | |  |  |  |  |  |  |  |  |  |  |  |  |
|  | Dutch Reformed | (ref.) |  |  | (ref.) |  |  | (ref.) |  |  | (ref.) |  |  |
|  | Other Protestant | 0.785 | 0.204 |  | 1.260 | 0.590 |  | 0.637 | 0.074 | * | 1.036 | 0.932 |  |
|  | Catholic | 1.174 | 0.302 |  | 2.008 | 0.059 | * | 0.877 | 0.524 |  | 1.700 | 0.116 |  |
|  | Jewish | 0.158 | 0.002 | *** | 0.854 | 0.842 |  | 0.097 | 0.016 | ** | - |  |  |
|  | Unknown | 1.064 | 0.871 |  | 1.224 | 0.798 |  | 0.903 | 0.832 |  | 1.568 | 0.678 |  |
|  |  |  |  |  |  |  |  |  |  |  |  |  |  |
| Social class | |  |  |  |  |  |  |  |  |  |  |  |  |
|  | Elite | 0.595 | 0.403 |  | 1.237 | 0.848 |  | 0.660 | 0.587 |  | - |  |  |
|  | Middle class | 1.177 | 0.375 |  | 1.353 | 0.451 |  | 1.033 | 0.893 |  | 1.364 | 0.453 |  |
|  | Skilled | 0.953 | 0.762 |  | 1.049 | 0.895 |  | 0.810 | 0.299 |  | 1.132 | 0.732 |  |
|  | Semi-skilled | 0.839 | 0.410 |  | 0.545 | 0.287 |  | 0.966 | 0.892 |  | 0.557 | 0.335 |  |
|  | Unskilled | (ref.) |  |  | (ref.) |  |  | (ref.) |  |  | (ref.) |  |  |
|  | Unknown | 0.746 | 0.390 |  | 1.376 | 0.594 |  | 0.653 | 0.348 |  | 0.278 | 0.067 | * |
|  |  |  |  |  |  |  |  |  |  |  |  |  |  |
| Single mother | | 4.011 | 0.000 | *** | 2.323 | 0.155 |  | 4.337 | 0.002 | *** | 14.243 | 0.000 | *** |
|  |  |  |  |  |  |  |  |  |  |  |  |  |  |
| Season of birth | |  |  |  |  |  |  |  |  |  |  |  |  |
|  | Spring | (ref.) |  |  | (ref.) |  |  | (ref.) |  |  | (ref.) |  |  |
|  | Summer | 0.752 | 0.088 | * | 1.055 | 0.891 |  | 0.668 | 0.050 | * | 0.833 | 0.722 |  |
|  | Autumn | 0.876 | 0.386 |  | 1.023 | 0.951 |  | 0.569 | 0.005 | *** | 3.196 | 0.003 | *** |
|  | Winter | 0.953 | 0.747 |  | 1.517 | 0.196 |  | 0.729 | 0.095 | * | 1.943 | 0.108 |  |
|  |  |  |  |  |  |  |  |  |  |  |  |  |  |
| Tax value | |  |  |  |  |  |  |  |  |  |  |  |  |
|  | <100 | (ref.) |  |  | (ref.) |  |  | (ref.) |  |  | (ref.) |  |  |
|  | 100-200 | 0.755 | 0.050 | * | 1.244 | 0.475 |  | 0.690 | 0.044 | ** | 0.630 | 0.197 |  |
|  | 200+ | 1.008 | 0.964 |  | 1.425 | 0.396 |  | 0.876 | 0.578 |  | 1.107 | 0.808 |  |
|  |  |  |  |  |  |  |  |  |  |  |  |  |  |
|  |  |  |  |  |  |  |  |  |  |  |  |  |  |
| Backstreet alley | | 0.734 | 0.097 | * | 1.049 | 0.898 |  | 0.696 | 0.140 |  | 0.707 | 0.439 |  |
|  |  |  |  |  |  |  |  |  |  |  |  |  |  |
| Distance to midwife | |  |  |  |  |  |  |  |  |  |  |  |  |
|  | <75 | (ref.) |  |  | (ref.) |  |  | (ref.) |  |  | (ref.) |  |  |
|  | 75-150 | 1.021 | 0.889 |  | 1.009 | 0.976 |  | 0.969 | 0.871 |  | 1.236 | 0.542 |  |
|  | 150-300 | 0.958 | 0.774 |  | 0.594 | 0.131 |  | 1.181 | 0.376 |  | 0.857 | 0.670 |  |
|  | 300-600 | 1.224 | 0.387 |  | 1.171 | 0.778 |  | 1.020 | 0.951 |  | 1.860 | 0.185 |  |
|  | 600+ | 1.573 | 0.420 |  | 2.611 | 0.540 |  | 0.981 | 0.975 |  | 6.562 | 0.190 |  |
|  |  |  |  |  |  |  |  |  |  |  |  |  |  |
|  |  |  |  |  |  |  |  |  |  |  |  |  |  |
| Water quality canals | |  |  |  |  |  |  |  |  |  |  |  |  |
|  | Seafront | (ref.) |  |  | (ref.) |  |  | (ref.) |  |  | (ref.) |  |  |
|  | "Best" | 1.188 | 0.652 |  | 1.160 | 0.896 |  | 1.408 | 0.456 |  | 0.430 | 0.429 |  |
|  | "Intermediate" | 1.269 | 0.214 |  | 1.142 | 0.755 |  | 1.434 | 0.163 |  | 0.941 | 0.879 |  |
|  | "Worst" | 1.652 | 0.058 | * | 3.662 | 0.028 | ** | 1.569 | 0.194 |  | 0.919 | 0.887 |  |
|  |  |  |  |  |  |  |  |  |  |  |  |  |  |
|  |  |  |  |  |  |  |  |  |  |  |  |  |  |
| House in front of canal | | 0.782 | 0.092 | * | 0.884 | 0.716 |  | 0.722 | 0.083 | * | 0.806 | 0.545 |  |
|  |  |  |  |  |  |  |  |  |  |  |  |  |  |
| Neighborhood | |  |  |  |  |  |  |  |  |  |  |  |  |
|  | Centre | (ref.) |  |  | (ref.) |  |  | (ref.) |  |  | (ref.) |  |  |
|  | Jewish quarter | 0.390 | 0.015 | ** | 0.437 | 0.258 |  | 0.230 | 0.014 | ** | 1.594 | 0.529 |  |
|  | Islands | 0.777 | 0.329 |  | 0.708 | 0.587 |  | 0.745 | 0.381 |  | 0.988 | 0.983 |  |
|  | Jordaan | 0.832 | 0.506 |  | 0.412 | 0.169 |  | 0.815 | 0.563 |  | 1.912 | 0.344 |  |
|  | Northern canals | 0.469 | 0.018 | ** | 0.641 | 0.513 |  | 0.292 | 0.010 | ** | 0.879 | 0.843 |  |
|  | Southern canals | 0.849 | 0.503 |  | 0.422 | 0.140 |  | 0.902 | 0.731 |  | 1.353 | 0.603 |  |
|  | Plantage Weesper | 1.293 | 0.249 |  | 1.250 | 0.646 |  | 0.970 | 0.923 |  | 3.024 | 0.021 | ** |
|  | Outer areas | 1.277 | 0.545 |  | 0.707 | 0.743 |  | 1.908 | 0.163 |  | 0.533 | 0.676 |  |
|  |  |  |  |  |  |  |  |  |  |  |  |  |  |

Notes: Neonatal = first 28 days, early post neonatal = from day 29 up to the 6^th^ month, later post neonatal = from the 7^th^ up to the 12^th^ month; significance * p<0.1, ** p<0.01, *** p<0.001; (ref.) = reference category; - = omitted variable categories.
Sources: Own calculations based on linked data from Amsterdam Cause-of-death Database, Amsterdam birth (1856) and death (1856-57) certificates, Amsterdam population register 1851-1853, and HISGIS Amsterdam.

Table S4. Fine-Gray competing risk model subdistribution hazard ratios (SHR) for infant mortality from convulsions in Amsterdam, children born in 1856

|  |  | Mulltivariate models | | | | | | | | | | | |
| --- | --- | --- | --- | --- | --- | --- | --- | --- | --- | --- | --- | --- | --- |
|  |  | Total | | | Neonatal | | | Early post neonatal | | | Later post neonatal | | |
|  |  | SHR | P>\|z\| |  | SHR | P>\|z\| |  | SHR | P>\|z\| |  | SHR | P>\|z\| |  |
|  |  |  |  |  |  |  |  |  |  |  |  |  |  |
| Sex | Males | 1.288 | 0.033 | ** | 1.613 | 0.039 | ** | 1.175 | 0.325 |  | 1.079 | 0.781 |  |
|  |  |  |  |  |  |  |  |  |  |  |  |  |  |
| Age mother | |  |  |  |  |  |  |  |  |  |  |  |  |
|  | 15-19 | 2.104 | 0.227 |  | - |  |  | 2.461 | 0.222 |  | - |  |  |
|  | 20-24 | (ref.) |  |  | (ref.) |  |  | (ref.) |  |  | (ref.) |  |  |
|  | 25-29 | 1.808 | 0.102 |  | 2.136 | 0.319 |  | 1.735 | 0.259 |  | 1.701 | 0.488 |  |
|  | 30-34 | 1.693 | 0.141 |  | 1.992 | 0.340 |  | 1.340 | 0.558 |  | 1.925 | 0.384 |  |
|  | 35-39 | 1.932 | 0.069 | * | 3.178 | 0.113 |  | 1.441 | 0.472 |  | 1.197 | 0.821 |  |
|  | 40+ | 2.222 | 0.044 | ** | 2.288 | 0.313 |  | 2.626 | 0.070 | * | 1.674 | 0.548 |  |
|  | Unknown | 1.759 | 0.392 |  | 2.020 | 0.597 |  | 4.210 | 0.129 |  | 0.367 | 0.488 |  |
|  |  |  |  |  |  |  |  |  |  |  |  |  |  |
| Age difference father | |  |  |  |  |  |  |  |  |  |  |  |  |
|  | Younger (2+ years) | 0.928 | 0.725 |  | 0.927 | 0.853 |  | 0.664 | 0.201 |  | 1.608 | 0.296 |  |
|  | Equal (-2 to +2 years) | 1.146 | 0.430 |  | 1.292 | 0.437 |  | 1.058 | 0.818 |  | 1.213 | 0.644 |  |
|  | Older (2-9 years) | (ref.) |  |  | (ref.) |  |  | (ref.) |  |  | (ref.) |  |  |
|  | Much older (9+ years) | 1.050 | 0.846 |  | 1.314 | 0.565 |  | 1.216 | 0.547 |  | 0.755 | 0.673 |  |
|  | Unknown | 1.328 | 0.512 |  | 1.892 | 0.435 |  | 0.550 | 0.383 |  | 2.962 | 0.189 |  |
|  |  |  |  |  |  |  |  |  |  |  |  |  |  |
| Multiple births | | 2.892 | 0.000 | *** | 4.290 | 0.000 | *** | 3.230 | 0.000 | *** | 3.833 | 0.010 | ** |
|  |  |  |  |  |  |  |  |  |  |  |  |  |  |
| Religion | |  |  |  |  |  |  |  |  |  |  |  |  |
|  | Dutch Reformed | (ref.) |  |  | (ref.) |  |  | (ref.) |  |  | (ref.) |  |  |
|  | Other Protestant | 0.722 | 0.132 |  | 1.120 | 0.760 |  | 0.767 | 0.378 |  | 0.331 | 0.074 | * |
|  | Catholic | 1.250 | 0.172 |  | 1.228 | 0.547 |  | 1.331 | 0.210 |  | 1.048 | 0.894 |  |
|  | Jewish | 0.792 | 0.440 |  | 1.356 | 0.577 |  | 0.606 | 0.236 |  | 0.454 | 0.370 |  |
|  | Unknown | 0.865 | 0.706 |  | 0.855 | 0.851 |  | 0.700 | 0.443 |  | 1.607 | 0.648 |  |
|  |  |  |  |  |  |  |  |  |  |  |  |  |  |
| Social class | |  |  |  |  |  |  |  |  |  |  |  |  |
|  | Elite | 0.917 | 0.868 |  | 0.902 | 0.924 |  | 0.347 | 0.291 |  | 1.942 | 0.549 |  |
|  | Middle class | 1.079 | 0.691 |  | 0.708 | 0.356 |  | 0.864 | 0.594 |  | 2.685 | 0.020 | ** |
|  | Skilled | 1.074 | 0.667 |  | 1.200 | 0.525 |  | 0.932 | 0.762 |  | 1.000 | 1.000 |  |
|  | Semi-skilled | 1.061 | 0.785 |  | 0.928 | 0.855 |  | 0.931 | 0.813 |  | 1.805 | 0.248 |  |
|  | Unskilled | (ref.) |  |  | (ref.) |  |  | (ref.) |  |  | (ref.) |  |  |
|  | Unknown | 1.010 | 0.978 |  | 0.675 | 0.579 |  | 1.020 | 0.967 |  | 1.449 | 0.732 |  |
|  |  |  |  |  |  |  |  |  |  |  |  |  |  |
| Single mother | | 1.894 | 0.124 |  | 1.711 | 0.515 |  | 2.653 | 0.067 | * | 1.949 | 0.563 |  |
|  |  |  |  |  |  |  |  |  |  |  |  |  |  |
| Season of birth | |  |  |  |  |  |  |  |  |  |  |  |  |
|  | Spring | (ref.) |  |  | (ref.) |  |  | (ref.) |  |  | (ref.) |  |  |
|  | Summer | 1.385 | 0.063 | * | 1.178 | 0.604 |  | 1.361 | 0.225 |  | 1.804 | 0.144 |  |
|  | Autumn | 1.297 | 0.145 |  | 0.939 | 0.856 |  | 1.559 | 0.073 | * | 1.251 | 0.617 |  |
|  | Winter | 1.538 | 0.011 | ** | 1.444 | 0.224 |  | 1.574 | 0.060 | * | 1.806 | 0.138 |  |
|  |  |  |  |  |  |  |  |  |  |  |  |  |  |
| Tax value | |  |  |  |  |  |  |  |  |  |  |  |  |
|  | <100 | (ref.) |  |  | (ref.) |  |  | (ref.) |  |  | (ref.) |  |  |
|  | 100-200 | 1.090 | 0.562 |  | 1.472 | 0.180 |  | 1.011 | 0.958 |  | 0.952 | 0.884 |  |
|  | 200+ | 1.131 | 0.515 |  | 1.665 | 0.176 |  | 1.042 | 0.875 |  | 0.959 | 0.923 |  |
|  |  |  |  |  |  |  |  |  |  |  |  |  |  |
|  |  |  |  |  |  |  |  |  |  |  |  |  |  |
| Backstreet alley | | 0.814 | 0.291 |  | 0.735 | 0.456 |  | 0.873 | 0.607 |  | 0.662 | 0.348 |  |
|  |  |  |  |  |  |  |  |  |  |  |  |  |  |
| Distance to midwife | |  |  |  |  |  |  |  |  |  |  |  |  |
|  | <75 | (ref.) |  |  | (ref.) |  |  | (ref.) |  |  | (ref.) |  |  |
|  | 75-150 | 0.639 | 0.008 | *** | 0.690 | 0.263 |  | 0.740 | 0.192 |  | 0.433 | 0.042 | ** |
|  | 150-300 | 0.908 | 0.529 |  | 0.955 | 0.877 |  | 0.857 | 0.489 |  | 1.029 | 0.931 |  |
|  | 300-600 | 1.057 | 0.840 |  | 0.414 | 0.230 |  | 1.190 | 0.622 |  | 2.689 | 0.086 | * |
|  | 600+ | 0.987 | 0.986 |  | 1.584 | 0.657 |  | 0.825 | 0.858 |  | - |  |  |
|  |  |  |  |  |  |  |  |  |  |  |  |  |  |
|  |  |  |  |  |  |  |  |  |  |  |  |  |  |
| Water quality canals | |  |  |  |  |  |  |  |  |  |  |  |  |
|  | Seafront | (ref.) |  |  | (ref.) |  |  | (ref.) |  |  | (ref.) |  |  |
|  | "Best" | 0.970 | 0.942 |  | 0.949 | 0.948 |  | 0.603 | 0.396 |  | 3.540 | 0.190 |  |
|  | "Intermediate" | 0.963 | 0.833 |  | 0.673 | 0.279 |  | 1.043 | 0.872 |  | 1.374 | 0.448 |  |
|  | "Worst" | 1.146 | 0.615 |  | 0.764 | 0.606 |  | 1.083 | 0.820 |  | 2.687 | 0.209 |  |
|  |  |  |  |  |  |  |  |  |  |  |  |  |  |
|  |  |  |  |  |  |  |  |  |  |  |  |  |  |
| House in front of canal | | 0.851 | 0.272 |  | 0.947 | 0.838 |  | 0.854 | 0.448 |  | 0.475 | 0.046 | ** |
|  |  |  |  |  |  |  |  |  |  |  |  |  |  |
| Neighborhood | |  |  |  |  |  |  |  |  |  |  |  |  |
|  | Centre | (ref.) |  |  | (ref.) |  |  | (ref.) |  |  | (ref.) |  |  |
|  | Jewish quarter | 2.092 | 0.006 | *** | 5.432 | 0.005 | *** | 1.948 | 0.056 | * | 0.678 | 0.621 |  |
|  | Islands | 1.055 | 0.844 |  | 3.116 | 0.059 | * | 1.136 | 0.720 |  | 0.167 | 0.037 | ** |
|  | Jordaan | 1.345 | 0.325 |  | 5.057 | 0.017 | ** | 1.089 | 0.828 |  | 0.506 | 0.378 |  |
|  | Northern canals | 0.863 | 0.637 |  | 1.049 | 0.958 |  | 0.898 | 0.790 |  | 0.527 | 0.390 |  |
|  | Southern canals | 1.415 | 0.210 |  | 2.604 | 0.129 |  | 1.699 | 0.126 |  | 0.530 | 0.381 |  |
|  | Plantage Weesper | 1.207 | 0.482 |  | 2.311 | 0.183 |  | 0.808 | 0.609 |  | 1.108 | 0.825 |  |
|  | Outer areas | 1.172 | 0.759 |  | 3.924 | 0.231 |  | 1.517 | 0.521 |  | 0.242 | 0.267 |  |
|  |  |  |  |  |  |  |  |  |  |  |  |  |  |

Notes: Neonatal = first 28 days, early post neonatal = from day 29 up to the 6^th^ month, later post neonatal = from the 7^th^ up to the 12^th^ month; significance * p<0.1, ** p<0.01, *** p<0.001; (ref.) = reference category; - = omitted variable categories.
Sources: Own calculations based on linked data from Amsterdam Cause-of-death Database, Amsterdam birth (1856) and death (1856-57) certificates, Amsterdam population register 1851-1853, and HISGIS Amsterdam.

Table S5. Fine-Gray competing risk model subdistribution hazard ratios (SHR) for infant mortality from unknown causes in Amsterdam, children born in 1856

|  |  | Mulltivariate models | | | | | | | | | | | |
| --- | --- | --- | --- | --- | --- | --- | --- | --- | --- | --- | --- | --- | --- |
|  |  | Total | | | Neonatal | | | Early post neonatal | | | Later post neonatal | | |
|  |  | SHR | P>\|z\| |  | SHR | P>\|z\| |  | SHR | P>\|z\| |  | SHR | P>\|z\| |  |
|  |  |  |  |  |  |  |  |  |  |  |  |  |  |
| Sex | Males | 1.060 | 0.577 |  | 1.070 | 0.779 |  | 0.986 | 0.916 |  | 1.369 | 0.190 |  |
|  |  |  |  |  |  |  |  |  |  |  |  |  |  |
| Age mother | |  |  |  |  |  |  |  |  |  |  |  |  |
|  | 15-19 | 1.051 | 0.926 |  | 0.770 | 0.820 |  | 0.912 | 0.898 |  | - |  |  |
|  | 20-24 | (ref.) |  |  | (ref.) |  |  | (ref.) |  |  | (ref.) |  |  |
|  | 25-29 | 0.917 | 0.766 |  | 0.294 | 0.048 | ** | 1.117 | 0.765 |  | 1.434 | 0.649 |  |
|  | 30-34 | 1.018 | 0.950 |  | 0.671 | 0.456 |  | 0.782 | 0.511 |  | 2.608 | 0.196 |  |
|  | 35-39 | 1.098 | 0.752 |  | 0.965 | 0.951 |  | 0.944 | 0.881 |  | 2.114 | 0.334 |  |
|  | 40+ | 1.407 | 0.276 |  | 0.944 | 0.926 |  | 1.410 | 0.402 |  | 2.253 | 0.326 |  |
|  | Unknown | 2.673 | 0.071 | * | 1.281 | 0.742 |  | 2.250 | 0.294 |  | - |  |  |
|  |  |  |  |  |  |  |  |  |  |  |  |  |  |
| Age difference father | |  |  |  |  |  |  |  |  |  |  |  |  |
|  | Younger (2+ years) | 1.008 | 0.965 |  | 1.866 | 0.195 |  | 0.951 | 0.846 |  | 0.711 | 0.411 |  |
|  | Equal (-2 to +2 years) | 0.983 | 0.918 |  | 1.625 | 0.285 |  | 1.009 | 0.968 |  | 0.676 | 0.293 |  |
|  | Older (2-9 years) | (ref.) |  |  | (ref.) |  |  | (ref.) |  |  | (ref.) |  |  |
|  | Much older (9+ years) | 1.093 | 0.690 |  | 2.134 | 0.164 |  | 0.866 | 0.648 |  | 1.249 | 0.617 |  |
|  | Unknown | 0.487 | 0.069 | * | 3.388 | 0.047 | ** | 0.361 | 0.074 | * | - |  |  |
|  |  |  |  |  |  |  |  |  |  |  |  |  |  |
| Multiple births | | 2.585 | 0.000 | *** | 8.176 | 0.000 | *** | 2.765 | 0.001 | *** | 0.844 | 0.865 |  |
|  |  |  |  |  |  |  |  |  |  |  |  |  |  |
| Religion | |  |  |  |  |  |  |  |  |  |  |  |  |
|  | Dutch Reformed | (ref.) |  |  | (ref.) |  |  | (ref.) |  |  | (ref.) |  |  |
|  | Other Protestant | 0.679 | 0.061 | * | 0.516 | 0.175 |  | 0.616 | 0.091 | * | 0.991 | 0.982 |  |
|  | Catholic | 1.016 | 0.924 |  | 0.599 | 0.249 |  | 1.087 | 0.693 |  | 1.182 | 0.647 |  |
|  | Jewish | 1.003 | 0.993 |  | 2.657 | 0.134 |  | 0.737 | 0.452 |  | 0.565 | 0.357 |  |
|  | Unknown | 0.971 | 0.936 |  | 0.336 | 0.057 | * | 1.454 | 0.492 |  | 1.607 | 0.651 |  |
|  |  |  |  |  |  |  |  |  |  |  |  |  |  |
| Social class | |  |  |  |  |  |  |  |  |  |  |  |  |
|  | Elite | 0.415 | 0.187 |  | - |  |  | 0.329 | 0.280 |  | 0.784 | 0.753 |  |
|  | Middle class | 0.696 | 0.042 | ** | 0.934 | 0.862 |  | 0.654 | 0.079 | * | 0.878 | 0.733 |  |
|  | Skilled | 0.821 | 0.180 |  | 0.981 | 0.953 |  | 0.823 | 0.322 |  | 0.743 | 0.372 |  |
|  | Semi-skilled | 1.061 | 0.755 |  | 1.447 | 0.359 |  | 0.991 | 0.970 |  | 1.036 | 0.938 |  |
|  | Unskilled | (ref.) |  |  | (ref.) |  |  | (ref.) |  |  | (ref.) |  |  |
|  | Unknown | 0.294 | 0.000 | *** | 0.344 | 0.008 | *** | 0.352 | 0.006 | *** | 0.201 | 0.150 |  |
|  |  |  |  |  |  |  |  |  |  |  |  |  |  |
| Single mother | | 10.224 | 0.000 | *** | 7.613 | 0.000 | *** | 9.615 | 0.000 | *** | 24.046 | 0.004 | *** |
|  |  |  |  |  |  |  |  |  |  |  |  |  |  |
| Season of birth | |  |  |  |  |  |  |  |  |  |  |  |  |
|  | Spring | (ref.) |  |  | (ref.) |  |  | (ref.) |  |  | (ref.) |  |  |
|  | Summer | 0.964 | 0.810 |  | 0.488 | 0.069 | * | 1.219 | 0.308 |  | 1.144 | 0.699 |  |
|  | Autumn | 1.184 | 0.235 |  | 1.142 | 0.650 |  | 1.282 | 0.192 |  | 0.833 | 0.602 |  |
|  | Winter | 1.058 | 0.698 |  | 0.936 | 0.832 |  | 1.116 | 0.571 |  | 1.201 | 0.581 |  |
|  |  |  |  |  |  |  |  |  |  |  |  |  |  |
| Tax value | |  |  |  |  |  |  |  |  |  |  |  |  |
|  | <100 | (ref.) |  |  | (ref.) |  |  | (ref.) |  |  | (ref.) |  |  |
|  | 100-200 | 0.889 | 0.376 |  | 0.955 | 0.878 |  | 0.953 | 0.779 |  | 0.905 | 0.739 |  |
|  | 200+ | 0.616 | 0.003 | *** | 0.618 | 0.251 |  | 0.668 | 0.055 | * | 0.537 | 0.101 |  |
|  |  |  |  |  |  |  |  |  |  |  |  |  |  |
|  |  |  |  |  |  |  |  |  |  |  |  |  |  |
| Backstreet alley | | 1.140 | 0.415 |  | 0.940 | 0.870 |  | 1.460 | 0.058 | * | 0.777 | 0.536 |  |
|  |  |  |  |  |  |  |  |  |  |  |  |  |  |
| Distance to midwife | |  |  |  |  |  |  |  |  |  |  |  |  |
|  | <75 | (ref.) |  |  | (ref.) |  |  | (ref.) |  |  | (ref.) |  |  |
|  | 75-150 | 1.086 | 0.559 |  | 2.484 | 0.018 | ** | 1.037 | 0.842 |  | 0.743 | 0.355 |  |
|  | 150-300 | 0.882 | 0.399 |  | 2.344 | 0.029 | ** | 0.793 | 0.237 |  | 0.784 | 0.431 |  |
|  | 300-600 | 1.131 | 0.612 |  | 2.735 | 0.054 | * | 0.970 | 0.928 |  | 1.028 | 0.959 |  |
|  | 600+ | 1.459 | 0.542 |  | 4.333 | 0.281 |  | 1.501 | 0.601 |  | 1.036 | 0.977 |  |
|  |  |  |  |  |  |  |  |  |  |  |  |  |  |
|  |  |  |  |  |  |  |  |  |  |  |  |  |  |
| Water quality canals | |  |  |  |  |  |  |  |  |  |  |  |  |
|  | Seafront | (ref.) |  |  | (ref.) |  |  | (ref.) |  |  | (ref.) |  |  |
|  | "Best" | 1.087 | 0.837 |  | 0.918 | 0.935 |  | 1.489 | 0.393 |  | 0.188 | 0.102 |  |
|  | "Intermediate" | 1.246 | 0.170 |  | 1.663 | 0.201 |  | 1.406 | 0.103 |  | 0.772 | 0.486 |  |
|  | "Worst" | 1.177 | 0.523 |  | 1.203 | 0.747 |  | 1.440 | 0.249 |  | 0.515 | 0.324 |  |
|  |  |  |  |  |  |  |  |  |  |  |  |  |  |
|  |  |  |  |  |  |  |  |  |  |  |  |  |  |
| House in front of canal | | 0.998 | 0.991 |  | 0.871 | 0.693 |  | 1.260 | 0.219 |  | 0.524 | 0.079 | * |
|  |  |  |  |  |  |  |  |  |  |  |  |  |  |
| Neighborhood | |  |  |  |  |  |  |  |  |  |  |  |  |
|  | Centre | (ref.) |  |  | (ref.) |  |  | (ref.) |  |  | (ref.) |  |  |
|  | Jewish quarter | 1.174 | 0.538 |  | 0.580 | 0.452 |  | 1.501 | 0.228 |  | 1.099 | 0.847 |  |
|  | Islands | 1.773 | 0.005 | *** | 3.459 | 0.007 | *** | 2.073 | 0.007 | *** | 0.643 | 0.374 |  |
|  | Jordaan | 0.976 | 0.933 |  | 2.415 | 0.178 |  | 0.774 | 0.480 |  | 1.116 | 0.876 |  |
|  | Northern canals | 0.437 | 0.027 | ** | 0.306 | 0.221 |  | 0.351 | 0.049 | ** | 0.930 | 0.905 |  |
|  | Southern canals | 0.794 | 0.393 |  | 0.540 | 0.413 |  | 0.998 | 0.995 |  | 0.563 | 0.421 |  |
|  | Plantage Weesper | 0.928 | 0.749 |  | 1.279 | 0.669 |  | 1.077 | 0.798 |  | 0.439 | 0.167 |  |
|  | Outer areas | 0.940 | 0.902 |  | 1.150 | 0.908 |  | 0.739 | 0.658 |  | 3.569 | 0.116 |  |
|  |  |  |  |  |  |  |  |  |  |  |  |  |  |

Notes: Neonatal = first 28 days, early post neonatal = from day 29 up to the 6^th^ month, later post neonatal = from the 7^th^ up to the 12^th^ month; significance * p<0.1, ** p<0.01, *** p<0.001; (ref.) = reference category; - = omitted variable categories.
Sources: Own calculations based on linked data from Amsterdam Cause-of-death Database, Amsterdam birth (1856) and death (1856-57) certificates, Amsterdam population register 1851-1853, and HISGIS Amsterdam.
